# Tropomyosin Tpm3.1 Is Required to Maintain the Structure and Function of the Axon Initial Segment

**DOI:** 10.1016/j.isci.2020.101053

**Published:** 2020-04-12

**Authors:** Amr Abouelezz, Holly Stefen, Mikael Segerstråle, David Micinski, Rimante Minkeviciene, Lauri Lahti, Edna C. Hardeman, Peter W. Gunning, Casper C. Hoogenraad, Tomi Taira, Thomas Fath, Pirta Hotulainen

**Affiliations:** 1 Minerva Foundation Institute for Medical Research, Biomedicum Helsinki 2U, Tukholmankatu 8, 00290 Helsinki, Finland; 2 HiLIFE - Neuroscience Center, University of Helsinki, Haartmaninkatu 8, 00290 Helsinki, Finland; 3 School of Medical Sciences, UNSW Sydney, Sydney, NSW 2052, Australia; 4 Faculty of Biological and Environmental Sciences, University of Helsinki, Viikinkaari 1, 00790 Helsinki, Finland; 5 Department of Computer Science, Aalto University School of Science, Espoo, Finland; 6 Cell Biology, Department of Biology, Faculty of Science, Utrecht University, Padualaan 8, 3584CH Utrecht, the Netherlands; 7 Faculty of Veterinary Medicine, University of Helsinki, Agnes Sjöbergin katu 2, 00790 Helsinki, Finland; 8 Dementia Research Centre, Faculty of Medicine and Health Sciences, Macquarie University, Sydney, NSW 2109, Australia

**Keywords:** Biological Sciences, Molecular Neuroscience, Cellular Neuroscience, Cell Biology

## Abstract

The axon initial segment (AIS) is the site of action potential initiation and serves as a cargo transport filter and diffusion barrier that helps maintain neuronal polarity. The AIS actin cytoskeleton comprises actin patches and periodic sub-membranous actin rings. We demonstrate that tropomyosin isoform Tpm3.1 co-localizes with actin patches and that the inhibition of Tpm3.1 led to a reduction in the density of actin patches. Furthermore, Tpm3.1 showed a periodic distribution similar to sub-membranous actin rings but Tpm3.1 was only partially congruent with sub-membranous actin rings. Nevertheless, the inhibition of Tpm3.1 affected the uniformity of the periodicity of actin rings. Furthermore, Tpm3.1 inhibition led to reduced accumulation of AIS structural and functional proteins, disruption in sorting somatodendritic and axonal proteins, and a reduction in firing frequency. These results show that Tpm3.1 is necessary for the structural and functional maintenance of the AIS.

## Highlights

• Tropomyosin isoform Tpm3.1 co-localizes with the actin cytoskeleton in the AIS
• Tpm3.1 inhibition led to a less organized AIS actin cytoskeleton
• Perturbation of Tpm3.1 function reduced the accumulation of AIS scaffolding proteins
• Tpm3.1 inhibition compromised cargo sorting and rapidly reduced firing frequency

## Graphical Abstract

**Figure:**
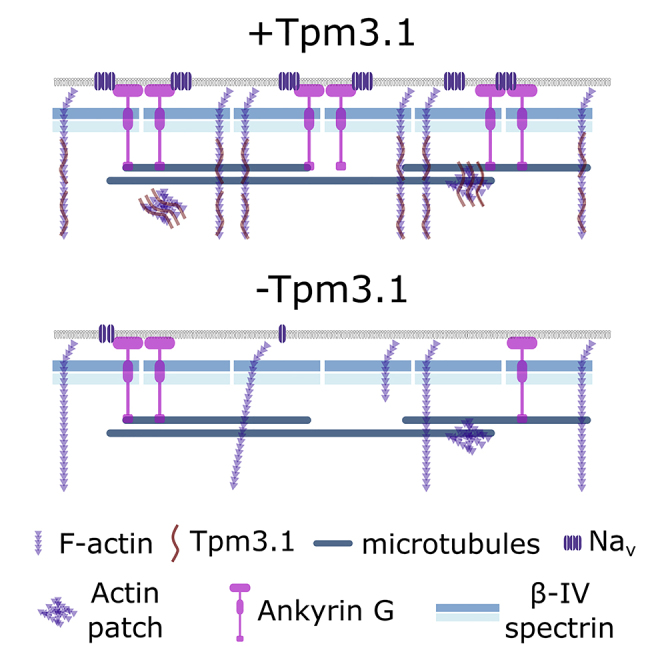

## Introduction

The proximal ends of axons in the vertebrate nervous system contain the axon initial segment (AIS). The AIS serves as the site of action potential initiation and plays a role in maintaining neuronal polarity. The clustering of sodium channels at the AIS facilitates spike generation (Kole et al., 2008), whereas its role in maintaining polarity is the result of a vesicle filter and diffusion barrier that restrict the entry of dendritic proteins and membrane lipids into the axon (Brachet et al., 2010, Nakada et al., 2003, Song et al., 2009, Sun et al., 2014, Winckler et al., 1999). The AIS is a remarkably stable structure comprising a specialized membrane and protein complex. Central to this complex is ankyrin G (Kordeli et al., 1995, Rasband, 2010), which acts as an adaptor that recruits other AIS proteins (Jenkins and Bennett, 2001); ankyrin G recruits and binds to βIV-spectrin (Yang et al., 2007), neurofascin-186 (NF-186) (Ango et al., 2004), as well as sodium (Zhou et al., 1998) and KCNQ2/3 channels (Pan et al., 2006). The loss of ankyrin G leads to the loss of all other AIS components (Hedstrom et al., 2008, Jenkins and Bennett, 2001, Zhou et al., 1998). The interaction of ankyrin G with microtubules (Freal et al., 2016, Kuijpers et al., 2016, Leterrier et al., 2011) and the binding of βIV-spectrin to actin filaments (Jenkins and Bennett, 2001, Leterrier et al., 2015) link the AIS complex to the cytoskeleton.

Although recent studies have shed light on the role of microtubules in establishing and maintaining the AIS (Freal et al., 2016, Klinman et al., 2017, Kuijpers et al., 2016, van Beuningen et al., 2015), the precise role of actin in the AIS remains unclear (Papandreou and Leterrier, 2018). Proper AIS development requires an intact actin cytoskeleton (Xu and Shrager, 2005), but the mature AIS is insensitive to actin-disrupting drugs (Abouelezz et al., 2019, Jones et al., 2014, Leterrier et al., 2015, Qu et al., 2017, Sanchez-Ponce et al., 2011, Song et al., 2009). This suggests that actin has no role in maintaining the structure of the AIS. Alternatively, actin filaments in the AIS may be resistant to the action of actin-disrupting drugs owing to a low rate of turnover. Nonetheless, the integrity of the actin cytoskeleton is important for the AIS vesicle filter and diffusion barrier (Al-Bassam et al., 2012, Nakada et al., 2003, Song et al., 2009, Winckler et al., 1999). Platinum replica electron microscopy showed that the AIS contains both short, stable actin filaments and longer, dynamic filaments (Jones et al., 2014). Actin-based myosin motors play a role in the targeted delivery of somatodendritic and axonal vesicles (Janssen et al., 2017, Lewis et al., 2009, Lewis et al., 2011), and actin filaments form patches in the AIS (Balasanyan et al., 2017, Watanabe et al., 2012) that may serve as cargo transport filters (Janssen et al., 2017, Leterrier and Dargent, 2014, Watanabe et al., 2012). Although actin patches are not exclusive to the AIS, AIS actin patches are more stable than patches along the distal axon; the washing away of diffuse molecules by cell permeabilization led to the loss of actin patches in the distal axon but not in the AIS (Watanabe et al., 2012). In addition, recent work showed that the actin-based non-muscle myosin II is involved in AIS structure and plasticity (Berger et al., 2018, Evans et al., 2017) and associates with axonal F-actin (Wang et al., 2020).

Super-resolution microscopy revealed the presence of periodic, sub-membranous, actin rings in the axon, forming a lattice with spectrin and ankyrin (D'Este et al., 2015, Leite et al., 2016, Leite and Sousa, 2016, Leterrier et al., 2015, Xu et al., 2013, Zhong et al., 2014). It was thought that axonal actin rings have similar molecular architecture than the erythrocyte membrane skeleton (Bennett and Baines, 2001, Leite et al., 2016, Leite and Sousa, 2016, Xu et al., 2013), where short, stable, adducin-capped actin filaments also form a sub-membranous lattice with spectrin and ankyrin (Fowler, 2013). Actin filaments in the erythrocyte membrane skeleton are also partly stabilized by tropomyosins (Fowler and Bennett, 1984, Sung et al., 2000, Sung and Lin, 1994). A recent study combining the super-resolution with platinum-replica electron microscopy, using mechanical unroofing of cells, brought new information of neuronal sub-membranous actin rings showing that actin rings are made of two long, intertwined actin filaments (Vassilopoulos et al., 2019).

Taken together, it is now well appreciated that the AIS contains two types of actin structures—patches and sub-membranous actin rings. Actin filaments forming actin rings are resistant to F-actin sequestering or severing drugs, suggesting they are stable (Abouelezz et al., 2019, Vassilopoulos et al., 2019). Currently, we lack information about the stability of F-actin in AIS actin patches. Furthermore, we know little about how the AIS actin cytoskeleton is regulated.

In this study, we show that the actin filaments in patches have a relatively slow turnover rate. The slow turnover rate of AIS actin filaments encouraged us to examine the potential role of tropomyosins. Tropomyosins are actin-binding proteins that form coiled-coil dimers and play a role in the regulation of the actin cytoskeleton in an isoform-specific manner (Gunning et al., 2008). Based on preliminary testing of available tropomyosin constructs, we focused studies on tropomyosin 3.1 (Tpm3.1). Tpm3.1 localizes to the axons of developing neurons and was suggested to play a role in neuronal polarity (Hannan et al., 1995, Vindin and Gunning, 2013, Weinberger et al., 1996). In addition, Tpm3.1 plays a role in regulating the filamentous actin pool in growth cones (Schevzov et al., 2008), growth cone motility (Fath et al., 2010), neurite branching (Schevzov et al., 2005), and neurite extension (Stefen et al., 2018). Tpm3.1 binds F-actin with high affinity (Gateva et al., 2017) and regulates the actions of key actin-binding proteins: (1) Tpm3.1 inhibits Arp2/3 complex-mediated polymerization (Kis-Bicskei et al., 2013); (2) Tpm3.1 enhances the phosphorylation of actin-depolymerizing factor/cofilin (Bryce et al., 2003) and inhibits the binding of cofilin to the pointed ends of actin filaments (Jansen and Goode, 2019), thus inhibiting filament severing as well as depolymerization at the pointed ends (Broschat, 1990, Jansen and Goode, 2019); (3) Tpm3.1 recruits tropomodulin to the pointed ends (Sung and Lin, 1994), further lowering the rate of depolymerization (Weber et al., 1994, Yamashiro et al., 2014); and (4) Tpm3.1 recruits and activates myosin II (Bryce et al., 2003, Gateva et al., 2017). Here, we show that tropomyosin 3.1 (Tpm3.1) is part of the AIS actin cytoskeleton and is necessary for the maintenance of AIS structure and function.

## Results

### The AIS Contains Patches of F-Actin with a Low Rate of Depolymerization

**Figure 1:**
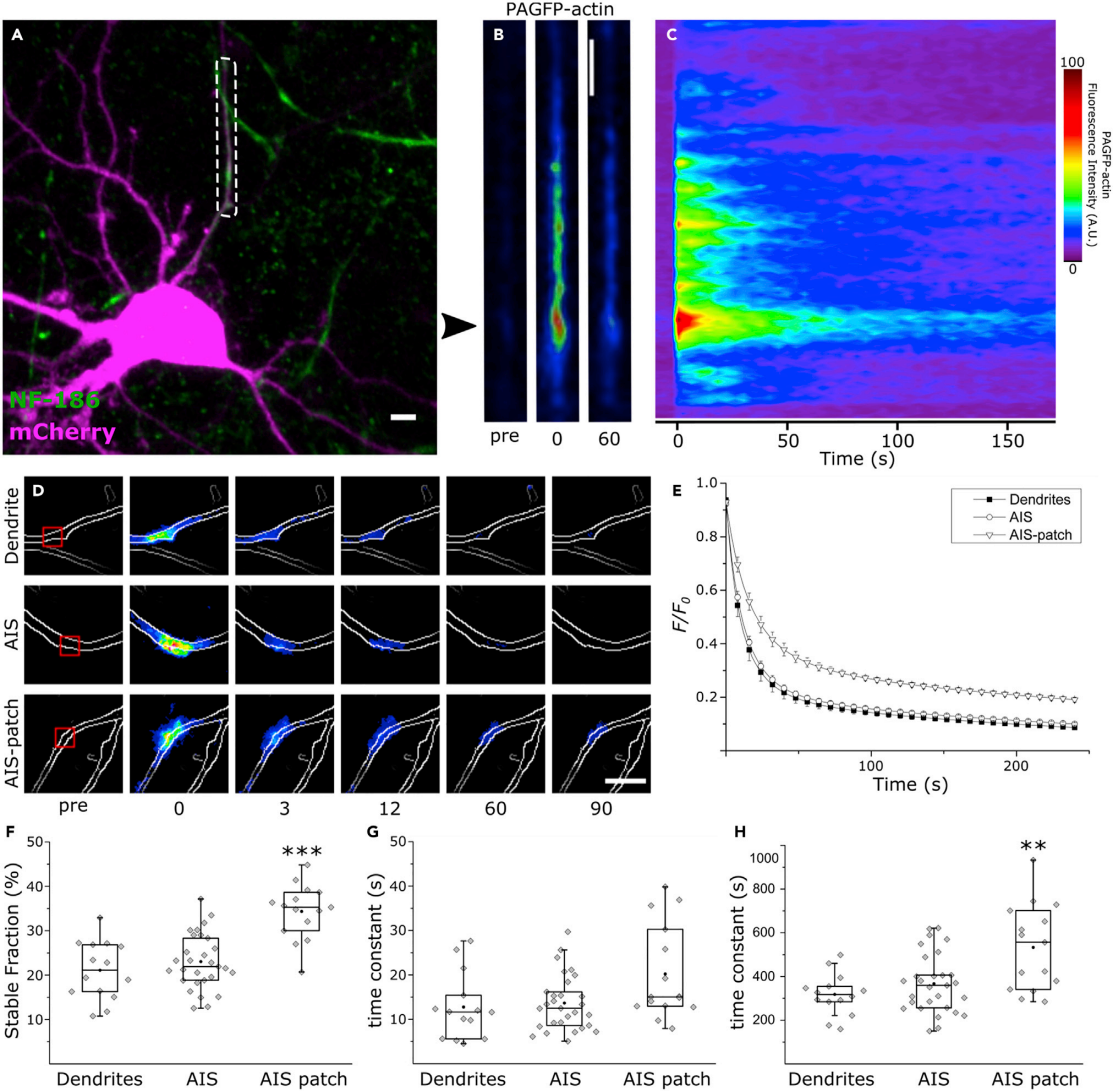
F-actin Patches in the AIS Have a Lower Rate of Depolymerization (A) We performed photoactivation within the dashed box representing the entire AIS in rat hippocampal neurons expressing mCherry and PAGFP-actin and monitored PAGFP fluorescence over time. PanNF186 served to label the AIS. (B) Higher magnification of the dashed box in (A) showing PAGFP-actin fluorescence 3 s before, immediately after, and 60 s after photoactivation. Arrowhead indicates F-actin patch. (C) PAGFP-actin fluorescence intensity profile along the AIS over time. (D) We performed photoactivation in a dendrite, the AIS, or an F-actin patch in the AIS (“AIS patch”). Photoactivation was limited to the small boxed region to enable a more accurate measurement of F-actin dynamics. Contour lines were constructed using mCherry fluorescence. (E) Average normalized fluorescence decay curve fits over time in dendrites, the AIS, and F-actin patches in the AIS. We fit fluorescence decay curves to a double-exponential decay function and compared the fitting parameters across groups. (F) Percentage of the stable fraction in dendrites, the AIS, and AIS actin patches (ANOVA, Tukey's test). (G) Time constants of the dynamic fractions (Mann-Whitney U test). (H) Time constants of the stable fractions (Mann-Whitney U test). Black circles represent mean value. Box borders represent the 25^th^ and 75^th^ percentiles, whiskers represent minimum and maximum values less than 1.5x the interquartile range lower or higher than the 25^th^ or 75^th^ percentiles, respectively (Tukey style). Dendrites: n = 14, 4 independent experiments; AIS: n = 29, 6 independent experiments; AIS patch: n = 15, 7 independent experiments. ∗ denotes statistical significance. ∗∗: p < 0.01; ∗∗∗: p < 0.001. Scale bar: 5 μm. See also Figure S1.

Previous studies have revealed that actin forms patches in the AIS (Balasanyan et al., 2017, D'Este et al., 2015, Janssen et al., 2017, Jones et al., 2014, Leite et al., 2016, Leite and Sousa, 2016, Leterrier et al., 2015, Watanabe et al., 2012, Xu et al., 2013, Zhong et al., 2014) that may play a role in the filtering of somatodendritic cargo (Balasanyan et al., 2017, Janssen et al., 2017, Watanabe et al., 2012). However, little is known about the dynamics and regulation of these actin patches. To investigate F-actin dynamics in the AIS, we expressed photoactivatable GFP-tagged actin (PAGFP-actin) in cultured rat hippocampal neurons (Koskinen and Hotulainen, 2014, Patterson and Lippincott-Schwartz, 2002). We co-transfected neurons at 8–10 days *in vitro* (DIV) using mCherry and PAGFP-actin and imaged them 40–56 h later. To label the AIS, we used an antibody against the extracellular domain of NF-186, 1–2 h before imaging (Hedstrom et al., 2008). To visualize the distribution of F-actin in the AIS, we applied a brief 405-nm laser pulse within a 30-μm-long region along the AIS (Figure 1A). The fluorescence intensity within this region was monitored for 3 min by capturing a frame every 3 s. Owing to the fast rate of diffusion of free actin monomers, the first frame taken after photoactivation (0 s) enables the visualization of only those monomers that were immobilized by incorporation into an actin filament (Honkura et al., 2008).

The distribution of F-actin in the AIS was uneven and a prominent patch under 1 μm in diameter showed a higher fluorescence intensity, corresponding to a higher concentration of F-actin (Figure 1B). Relative to the rest of the AIS, this actin patch was also the most long-lived (Figure 1C). To measure the rate of depolymerization more accurately, we confined the photoactivation to a square area roughly 5 μm^2^ in size (Figure 1D, red box). In addition to allowing for faster photoactivation, minimizing the area of photoactivation also minimizes the interference of photoactivated monomers that are incorporated into neighboring filaments after dissociation, leading to improved accuracy. Photoactivation was carried out within an AIS actin patch, in the AIS outside actin patches, and in a comparable dendritic segment that does not contain dendritic spines or branching points. An image was taken every 3 s and fluorescence intensity values were recorded. After subtracting the background fluorescence, we normalized the intensity values to the value at 0 s to obtain a normalized fluorescence decay curve. A double-exponential decay function gave the best fit for the decay curves in all groups (Koskinen and Hotulainen, 2014), indicating the presence of two pools of actin filaments with different rates of depolymerization. Accordingly, we fit the fluorescence decay curves to a double-exponential decay function (Figure 1E) and the fitting parameters were compared across groups.

The average proportion of the stable fraction of actin filaments (Figure 1F) was not significantly different between dendrites (21.1 ± 1.8%, mean ± SEM, n = 14, 4 independent experiments) and regions in the AIS outside the patches (23.0 ± 1.2%, mean ± SEM, n = 29, 6 independent experiments). Actin patches, however, had a higher proportion of stable filaments (34.4 ± 1.6%, mean ± SEM, n = 15, 7 independent experiments, p < 0.001, ANOVA, Tukey's test). In contrast, using the same experimental setup we found the proportion of stable actin filaments in dendritic spines to be 18% and 30% in cultured hippocampal neurons at 14 and 21 DIV, respectively (Koskinen et al., 2014). Figures 1G and 1H show the time constants for the dynamic and the stable pools of actin filaments, respectively. The average decay time constant of the dynamic pool was not significantly different between dendrites (12.76 ± 1.99 s, mean ± SEM, n = 14), regions in the AIS outside the patches (13.66 ± 1.16 s, mean ± SEM, n = 29), and actin patches (20.2 ± 2.74 s, mean ± SEM, n = 15, Mann-Whitney U test, Bonferroni corrected). The average decay time constant for the stable pool was not significantly different between dendrites (318 ± 25 s, mean ± SEM, n = 14) and regions in the AIS outside the patch (367 ± 25 s, mean ± SEM, n = 29). This value was higher in the actin patches (533 ± 50 s, mean ± SEM, n = 15, p < 0.01, Mann-Whitney U test, Bonferroni corrected) indicating that filaments in the AIS actin patches have a lower rate of depolymerization. It is likely that sub-membranous actin rings in the AIS had a minimal contribution to the readout in these experiments. This is partly due to the relatively low amount of F-actin in the sub-membranous lattice compared with intracellular F-actin within the area activated (10-fold less overall F-actin, Figure S1). In addition, actin filaments in the sub-membranous rings are relatively stable (Abouelezz et al., 2019, Vassilopoulos et al., 2019), possibly leading to a low rate of incorporation of PAGFP-actin monomers available for photoactivation. These data indicate that the AIS contains patches of F-actin that have a high proportion of stable filaments with a low rate of depolymerization.

### Tropomyosin Isoform Tpm3.1 Co-localizes with Actin Patches with Slow Turnover Rate

**Figure 2:**
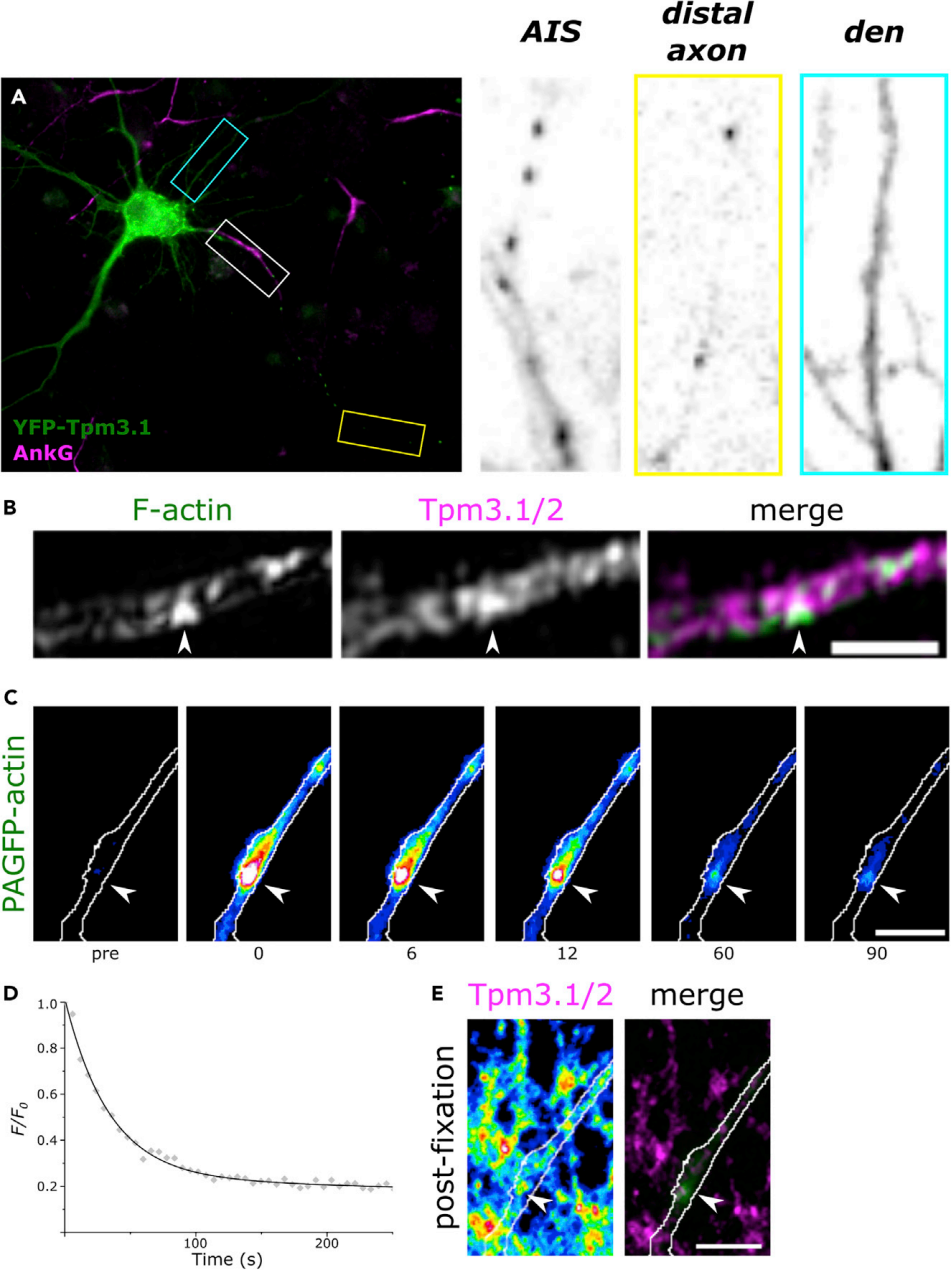
Tropomyosin Tpm3.1 Decorates Actin Patches in the AIS (A) Rat hippocampal neuron expressing YFP-Tpm3.1. Neurons were fixed 8 h post transfection. Anti-ankyrin G served to label the AIS. Patches of YFP-Tpm3.1 can be seen in the AIS (white box) and distally in the axon (yellow box), whereas the somatodendritic domain (cyan box) shows a diffuse, less intense distribution. (B) Maximum intensity projection of 3D-SIM reconstructions for F-actin and Tpm3.1/2. Arrowheads indicate actin patch. Scale bar: 1 μm. (C) Actin patch visualized in live hippocampal neuron using PAGFP-actin before photoactivation (pre), immediately after activation (0 s), and the time-points indicated in seconds. Arrowheads indicate actin patch. (D) Fluorescence decay over time (gray diamonds) of the actin patch in (C) and a double-exponential decay fit (solid black line). (E) Tpm3.1/2 distribution visualized using anti-γ/9d in the same area after fixation in 4% PFA. The intensity of Tpm3.1/2 immunofluorescence was higher in the region corresponding to the actin patch visualized in (C) (arrowhead). Scale bar: 5 μm. See also Figure S2.

The slow depolymerization rate suggests that AIS actin patches either contain relatively long actin filaments or are stabilized by actin-binding proteins. Tropomyosins are a family of actin-binding proteins that align along actin filaments and affect their characteristics. In neurons, altogether 12 isoforms are expressed (Gray et al., 2017, Abouelezz et al., 2019, Al-Bassam et al., 2012, Ango et al., 2004, Bach et al., 2009, Balasanyan et al., 2017) from *Tpm1*, *Tpm3*, and *Tpm4* genes. We examined the localization of available tropomyosin constructs, and from tested constructs, only Tpm3.1 was present in the AIS. YFP-Tpm3.1 expression showed bright puncta in the AIS and the distal axon and a diffuse distribution in the somatodendritic domain (Figure 2A). In contrast, the expression of the closely related YFP-tagged tropomyosin isoform Tpm3.2 (sharing 7 of 8 exons) showed a faint and diffuse distribution in the AIS and distal axon and mild enrichment in dendritic spines (Figure S2A). Therefore, we focused our further experiments on Tpm3.1.

We next wanted to test whether endogenous Tpm3.1 shows a similar staining pattern as YFP-Tpm3.1 and whether Tpm3.1 patches co-localize with actin patches. Epifluorescence imaging of anti-Tpm3.1/2 antibody γ/9d-staining showed patchy labeling (Figure S2B). Super-resolution structured illumination microscopy (SIM) imaging of phalloidin and γ/9d-antibody staining distribution revealed that patchy distribution of F-actin and Tpm3.1 co-localized (Figure 2B). γ/9d-antibody recognizes both Tpm3.1 and Tpm3.2, but as Tpm3.2 was absent from the AIS, we assumed that the fluorescence intensity detected using this antibody in the AIS originates from binding of Tpm3.1. Furthermore, we used PAGFP-actin to locate an actin patch with a low rate of depolymerization, then used anti-γ/9d to examine Tpm3.1 distribution. Tpm3.1 immunofluorescence showed a high intensity at the actin patch with slow PAGFP-actin fluorescence decay (Figure 2C, arrowheads), indicating that Tpm3.1 colocalizes with actin patches in the AIS (Figure 2E). Conversely, Tpm3.1 patches were resistant to detergent extraction (Figure S2C), indicating cytoskeletal association. On average, the intensity of Tpm3.1 was 2.5 times higher in patches than in the AIS overall, including patches (mean ± SEM: 2.52 ± 0.18, n = 25, 5 independent experiments). Based on these data, we conclude that Tpm3.1 is present in the AIS and co-localizes with actin patches.

### Tropomyosin Isoform Tpm3.1/2 Is Part of the Periodic AIS Actin Cytoskeleton

**Figure 3:**
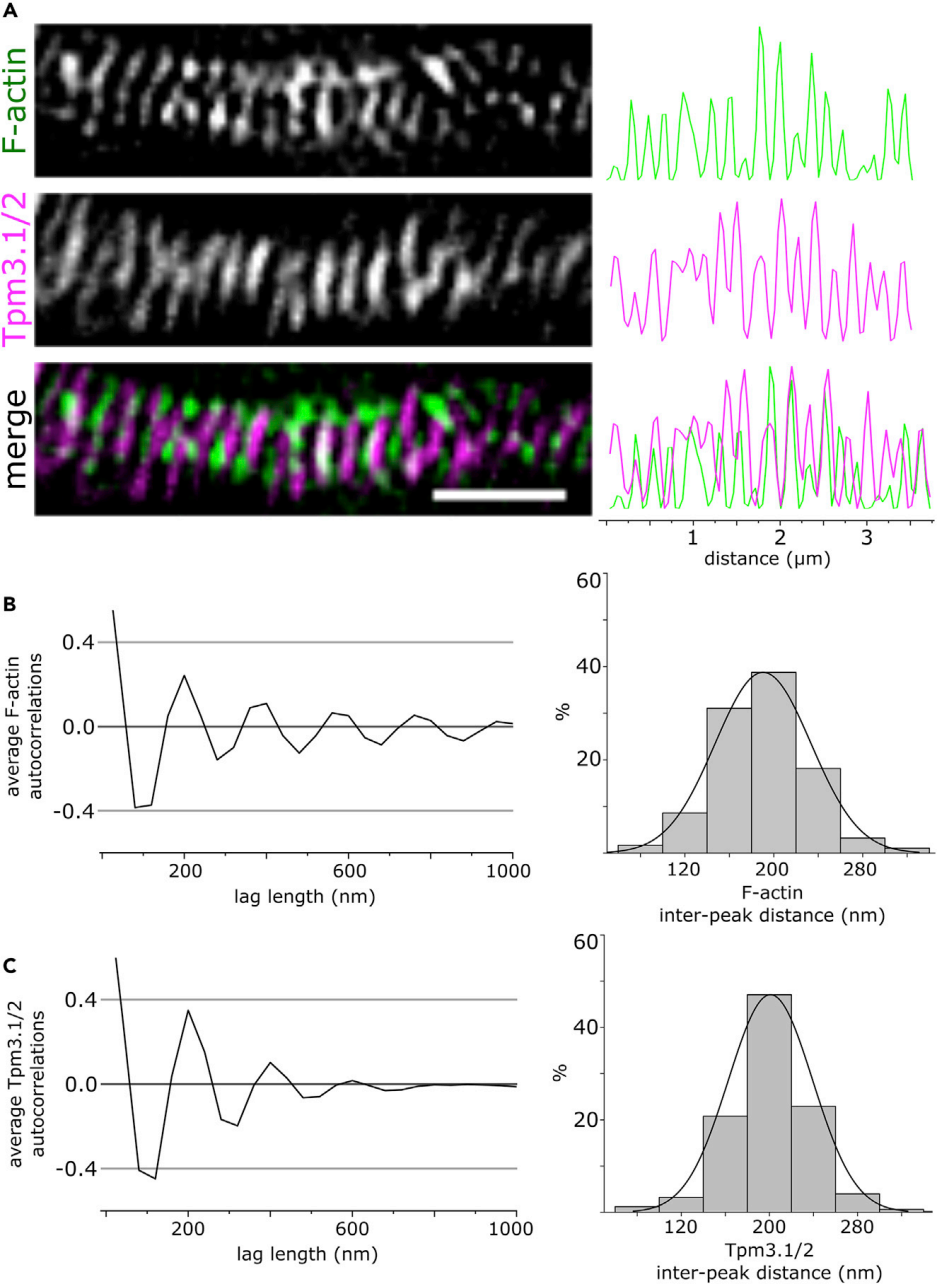
Tropomyosin Isoform Tpm3.1 Forms a Periodic Structure in the AIS (A) SIM reconstruction of the AIS of a rat hippocampal neuron at 14 DIV labeled using anti-γ/9d and Alexa 488-tagged phalloidin to visualize Tpm3.1 and F-actin, respectively. Anti-Ankyrin G served to label the AIS. Tpm3.1/2 shows a periodic structure partially corresponding to actin rings in the AIS. Right: fluorescence intensity profile along the AIS. (B) Left: Average autocorrelation of normalized phalloidin fluorescence intensity profiles showing autocorrelation at 200 nm. Right: Distance between individual peaks in normalized phalloidin fluorescence intensity profiles. About 37.7% of the peaks were separated by 200 nm. (C) Left: Average autocorrelation of normalized anti-γ/9d fluorescence intensity profiles showing autocorrelation at 200 nm. Right: Distance between individual peaks in normalized anti-γ/9d fluorescence intensity profiles. About 47.1% of the peaks were separated by 200 nm. n = 25 cells, 4 independent experiments. Scale bar: 1 μm. See also Figures S3 and S4.

In addition to patches, actin filaments in the AIS organize into periodic, sub-membranous rings, forming a lattice with spectrin and ankyrin (D'Este et al., 2015, Leite et al., 2016, Leite and Sousa, 2016, Leterrier et al., 2015, Xu et al., 2013, Zhong et al., 2014). We used SIM to test if Tpm3.1 also decorated actin filaments in these sub-membranous rings in cultured rat hippocampal neurons at 14 DIV. We used anti-γ/9d and Alexa 488-tagged phalloidin to visualize Tpm3.1 and F-actin, respectively. Anti-ankyrin G served to label the AIS. We optimized imaging parameters to visualize anti-γ/9d, tagged using Alexa 647. Similar to sub-membranous F-actin, Tpm3.1 showed a periodic distribution in the AIS (Figure 3A). To quantify the periodicity of Tpm3.1, we plotted fluorescence intensity profiles along regions in the AIS with visible periodicity and calculated the autocorrelation function for each profile. The average autocorrelation for the profiles measured showed an autocorrelation peak at a lag of 200 nm for both F-actin and Tpm3.1 (Figures 3B and 3C, left panels). Owing to the pixel size of the camera used (40 nm), the distances recorded are multiples of 40. Accordingly, a lag of 200 nm corresponds to the ∼190 nm reported earlier for actin rings and other components of the AIS sub-membranous lattice (D'Este et al., 2015, Leite et al., 2016, Leterrier et al., 2015, Xu et al., 2013, Zhong et al., 2014). For both F-actin and Tpm3.1, we measured the distance between individual neighboring fluorescence intensity peaks in each profile (Figures 3B and 3C, right panels). The mean inter-peak distance for F-actin was 190.36 ± 1.7 nm (mean ± SEM), with 37.7% of the profiles 200 nm apart. The mean inter-peak distance for Tpm3.1 was 200.98 ± 1.5 nm (mean ± SEM), with 47.1% of the distances measuring 200 nm. In distal axons and dendrites, Tpm3.1 showed less uniform periodicity compared with the AIS (Figure S3). Despite the similar inter-peak distance between F-actin and Tpm3.1 in the AIS, their distribution was only partially overlapping (Figure S4). Taken together, Tpm3.1 shows similar periodicity with sub-membranous F-actin rings. Tpm3.1 periodicity is most uniform in the AIS, whereas dendrites and distal regions of axons show less strict periodicity. The partial overlap of actin and Tpm3.1 suggests that Tpm3.1 is linked to sub-membranous actin rings but actin ring filaments are not necessarily aligned with Tpm3.1.

### Tpm3.1 Is Required for Maintaining the Structure of the AIS

We next examined the consequences of the perturbation of Tpm3.1 function for AIS structure. We used two distinct, well-characterized, small-molecule Tpm3.1 inhibitors, namely, TR100 (Bonello et al., 2016, Kee et al., 2015, Kee et al., 2018, Stehn et al., 2013) and Anisina (ATM3507) (Currier et al., 2017, Janco et al., 2019, Stehn et al., 2016) to inhibit Tpm3.1 and examined the accumulation of ankyrin G and other AIS markers at the AIS in mature cultured rat hippocampal neurons. TR100-mediated inhibition of Tpm3.1 does not inhibit the binding of Tpm3.1 to actin filaments but negates its effect on the rate of depolymerization (Bonello et al., 2016). The effects of TR100 on glucose-stimulated insulin secretion from the pancreatic islets were not seen in Tpm3.1 knockout mice, indicating that the impact of TR100 on glucose-stimulated insulin secretion was on-target (Kee et al., 2018). Similarly, Anisina (ATM3507) incorporates into the four-helix coiled coil overlap junction of the Tpm3.1 dimer as it binds to actin filaments, changing its lateral movement along the actin filament and altering the interactions of the actin filament with actin-binding proteins and myosin motors (Janco et al., 2019).

**Figure 4:**
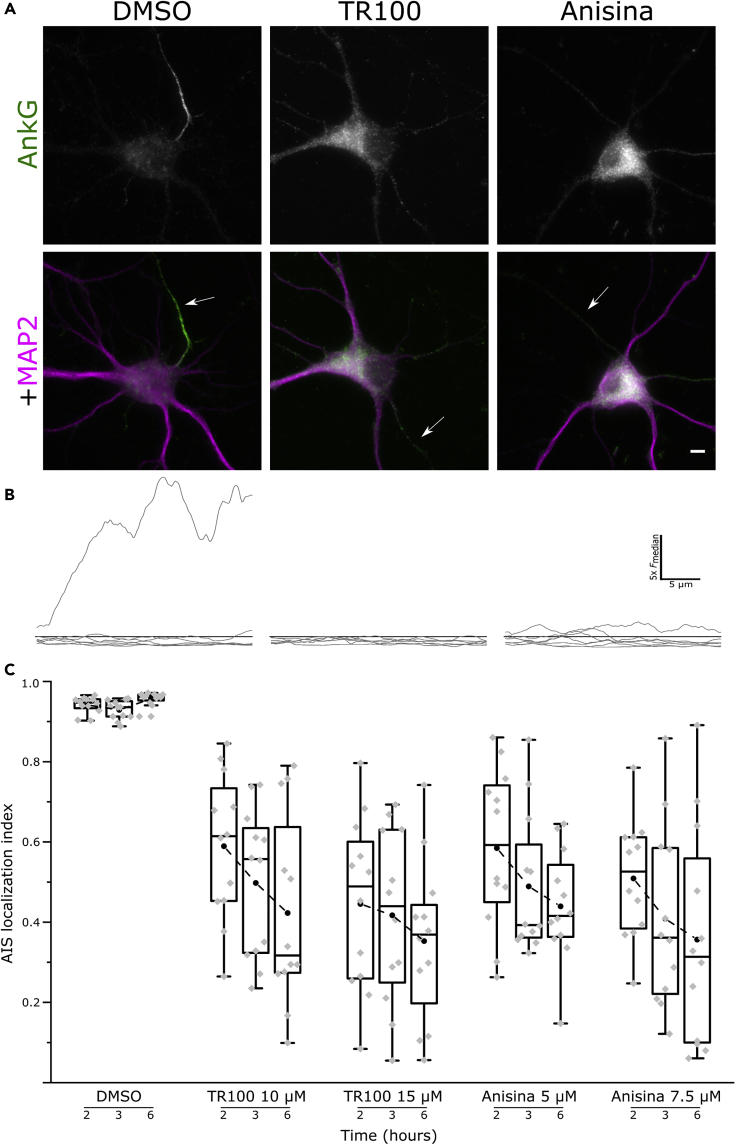
Inhibition of Tpm3.1 Reduces the Accumulation of Ankyrin G at the AIS (A) Rat hippocampal neurons treated at 10 DIV using DMSO or the small-molecule Tpm3.1 inhibitors TR100 or Anisina (ATM3507) for 2, 3, or 6 h. Anti-MAP2 served to label the somatodendritic domain; anti-ankyrin G served to measure the accumulation of ankyrin G. Arrows point to axons. (B) Smoothed ankyrin G fluorescence intensity line profiles (gray lines) along each neurite of the corresponding cell in (A), normalized to the median peak value (black line). (C) AIS localization indices for neurons treated using DMSO, TR100 (10 or 15 μM), or Anisina (5 or 7.5 μM) for 2, 3, or 6 h. All treatment groups were significantly different from DMSO controls (Mann-Whitney U test; DMSO 0.2%, 2 h: 0.94 ± 0.006, mean ± SEM; DMSO 0.2%, 3 h: 0.93 ± 0.006; DMSO 0.2%, 6 h: 0.96 ± 0.005; TR100 10 μM, 2 h: 0.59 ± 0.050, p < 0.001; TR100 10 μM, 3 h: 0.50 ± 0.051, p < 0.001; TR100 10 μM, 6 h: 0.42 ± 0.066, p < 0.001; TR100 15 μM, 2 h: 0.45 ± 0.060, p < 0.001; TR100 15 μM, 3 h: 0.42 ± 0.060, p < 0.001; TR100 15 μM, 6 h: 0.35 ± 0.056, p < 0.001; Anisina 5 μM, 2 h: 0.58 ± 0.055, p < 0.001; Anisina 5 μM, 3 h: 0.49 ± 0.049, p < 0.001; Anisina 5 μM, 6 h: 0.44 ± 0.039, p < 0.001; Anisina 7.5 μM, 2 h: 0.51 ± 0.041, p < 0.001; Anisina 7.5 μM, 3 h: 0.41 ± 0.062, p < 0.001; Anisina 7.5 μM, 6 h: 0.36 ± 0.075, p < 0.001; for each treatment, n = 12, 3 independent experiments). The mean ALI of the treatment groups was negatively correlated with treatment duration and concentration. Black circles represent mean values. Box borders represent the 25^th^ and 75^th^ percentiles, whiskers represent minimum and maximum values less than 1.5x the interquartile range lower or higher than the 25^th^ or 75^th^ percentiles, respectively (Tukey style). Dotted lines connect mean values. Scale bar: 5 μm. See also Figure S5.

We incubated sparse cultures of rat hippocampal neurons at 10 DIV using DMSO (0.2%), TR100 (10 or 15 μM), or Anisina (5 or 7.5 μM) for 2, 3, or 6 h and then used anti-ankyrin G to visualize the distribution of ankyrin G. Anti-MAP2 served to label the somatodendritic domain. Both TR100- and Anisina-mediated Tpm3.1 inhibition led to a notable reduction in the accumulation of ankyrin G at the AIS (Figure 4A). To quantify the reduction in ankyrin G accumulation, we blindly traced the fluorescence intensity profile of ankyrin G along the initial 30 μm of every neurite to calculate an AIS localization index (ALI, see Transparent Methods) (Figure 4B). For all treatment conditions, the ALI was lower than the corresponding DMSO controls (Figure 4C). The mean ALI was negatively correlated with the concentration of both TR100 (Pearson's coefficient; 2 h: −1.0; 3 h: −0.98; 6 h: −0.97) and Anisina (Pearson's coefficient; 2 h: −0.99; 3 h: −0.98; 6 h: −0.98), as well as with the duration of the treatment (Pearson's coefficient; TR100 10 μM: −0.94; TR100 15 μM: −1.0; Anisina 5 μM: −0.90; Anisina 7.5 μM: −0.90), suggesting dose and time dependence. We similarly observed a reduction in the mean ALI of ankyrin G after overnight inhibition of Tpm3.1 (Figures S5A and S5B). Tpm3.1 inhibition also abolished the accumulation at the AIS of all other AIS markers tested, namely, TRIM46, EB1, and neurofascin-186 (Figures S5C–S5F).

Although the loss of AIS structure upon Tpm3.1 inhibition using either TR100 or Anisina was clear, it is possible that this was a secondary effect rising from a general decay in neuronal health rather than a direct effect of the perturbation of Tpm3.1 function. In fact, oxidative stress (Clark et al., 2017) and neuronal injury (Schafer et al., 2009) resulted in the disruption of the AIS in a calpain-mediated manner. Schafer et al. (2009) showed that neuronal injury induced irreversible AIS disassembly through calpain-mediated proteolysis of ankyrin G and βIV-spectrin. However, this process was blocked by using the calpain inhibitor MDL28170. Thus, to test whether a similar calpain-dependent mechanism contributes to the loss of the AIS upon Tpm3.1 inhibition, we added calpain inhibitors to TR100 or Anisina treatments and measured the ALI of ankyrin G. Indeed, the loss of AIS structure upon Tpm3.1 inhibition was calpain independent; the mean ALI for all treatment conditions did not significantly change in the presence of 100 μM of the calpain inhibitor MDL28170 for the entire duration of the treatment (Figure S5G).

**Figure 5:**
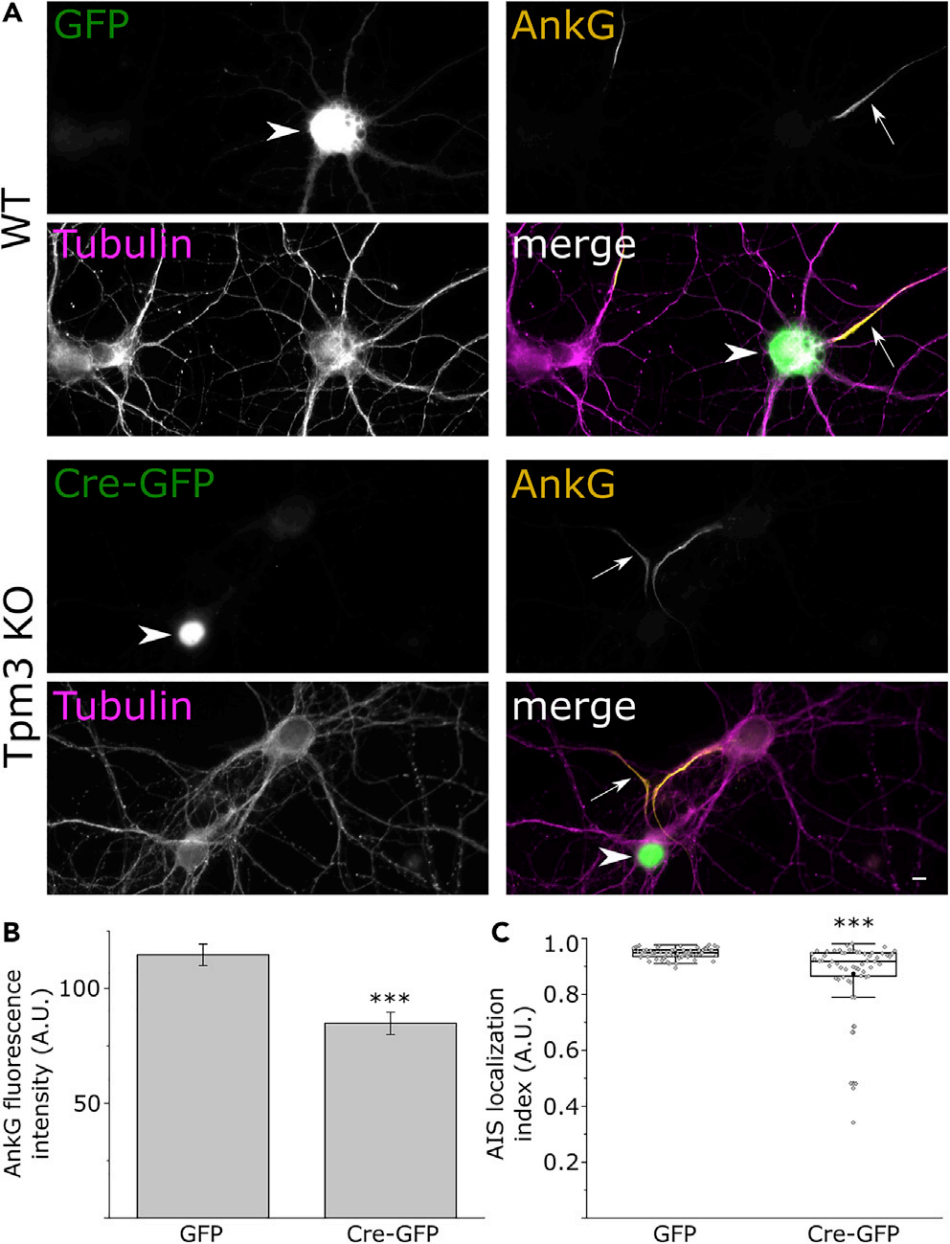
*Tpm3* Conditional Knockout Neurons Show a Reduced Accumulation of Ankyrin G at the AIS (A) Ankyrin G immunofluorescence for GFP- (wild-type, WT) and Cre GFP-expressing (*Tpm3* KO) neurons. β3-Tubulin was used to label neurons. Arrowheads indicate transfected neurons; arrows indicate axons of transfected neuron. Scale bar: 5 μm. (B) Average relative ankyrin G fluorescence intensity for each group (Mann-Whitney U test). Columns represent mean values. Error bars represent standard error of mean. GFP: n = 63, 3 independent experiments; Cre-GFP: n = 61, 3 independent experiments. ∗ denotes statistical significance. ∗∗∗: p < 0.001. (C) AIS localization indices (ALI) for neurons infected with GFP or Cre-GFP. The ALI of Cre-GFP infected neurons (0.87 ± 0.02, mean ± SEM, n = 57, 3 independent experiments) was lower than that of GFP-infected controls (0.95 ± 0.002, mean ± SEM, n = 61, 3 independent experiment, p < 0.001, Mann-Whitney U test). Black circles represent mean values. Box borders represent the 25^th^ and 75^th^ percentiles, whiskers represent minimum and maximum values less than 1.5x the interquartile range lower or higher than the 25^th^ or 75^th^ percentiles, respectively (Tukey style). See also Figures S6–S9.

To verify that the loss of ankyrin G accumulation at the AIS is a result of the loss of Tpm3.1 function, we generated a conditional knockout mouse model (Tp9 line, Figure S6, See Transparent Methods) for all Tpm3 isoforms containing exon 1b (namely, Tpm3.1, Tpm3.2, Tpm3.3, Tpm3.4, Tpm3.5, Tpm3.7, Tpm3.8, Tpm3.9, Tpm3.12, and Tpm3.13). We plated dissociated hippocampal neurons from these mice onto PDL-coated coverslips. To induce protein depletion, we transduced cultures using CMV-EGFP-Cre adeno-associated viruses (UNC Vector Core Facility). This resulted in a 10% transduction efficiency leading to a mixture of wild-type neurons and neurons with reduced Tpm3 level. Alternatively, we used CMV-EGFP adeno-associated viruses to transduce GFP—but not Cre—expression in sister cultures as controls. We confirmed the reduction of Tpm3 isoforms through immunostaining using an anti-Tpm3 antibody 2G10.2 (Figure S7). We then used anti-ankyrin G to visualize the accumulation of ankyrin G at the AIS. We first analyzed the intensity of ankyrin-G staining in GFP- or Cre-GFP-transfected cells (Figure 5B), and additionally, we analyzed the ALI as we did for TR100- and Anisina-treated cells (Figure 5C). The effect in cells from the conditional Tpm3 KO line with Cre expression was milder than that observed with the acute small-molecule inhibition. This milder effect is likely the result of residual Tpm3.1/2 present at the time of analysis.

As tropomyosins are a large family containing numerous isoforms obtained by alternative splicing from four different genes, we wanted to test whether the effect on ankyrin G accumulation at the AIS was tropomyosin gene specific or if knocking out any *Tpm* gene will lead to the same effect. Therefore, we repeated the experiment with conventional *Tpm4* knockout mice (Tp16 line) (Pleines et al., 2017). Neurons dissociated from this mouse line did not show any detectable difference in the accumulation of ankyrin G at the AIS (Figure S8). These data indicate that tropomyosin-dependent accumulation of ankyrin G at the AIS is *Tpm3* gene specific.

Furthermore, we obtained similar results by expressing short hairpin RNA (shRNA) specific to exon 9d for 4 days, which only depletes *Tpm3* isoforms Tpm3.1 and Tpm3.2 (Figures S9A–S9C). Similar to experiments carried out with conditional *Tpm3* knockout cells, Tpm3.1/2 protein level was only partially reduced, leading to an effect milder than that seen with acute inhibition of Tpm3.1. However, this effect was rescued by over-expressing YFP-tagged human Tpm3.1, which differs in sequence within the shRNA-targeting region, implicating that the shRNA-induced effect was indeed dependent on Tpm3.1 expression level (Figures S9D and S9E). Thus, we conclude that both pharmacological inhibition and genetic depletion of Tpm3.1 cause a notable defect in AIS structure. Together, these data suggest that the reduced accumulation of ankyrin G at the AIS is the result of the loss of Tpm3.1 function, indicating that Tpm3.1 is necessary for maintaining the structure of the AIS.

### Tpm3.1 Is Necessary for Maintaining the Selectivity of Axonal Transport and Sodium Channel Clustering at the AIS

**Figure 6:**
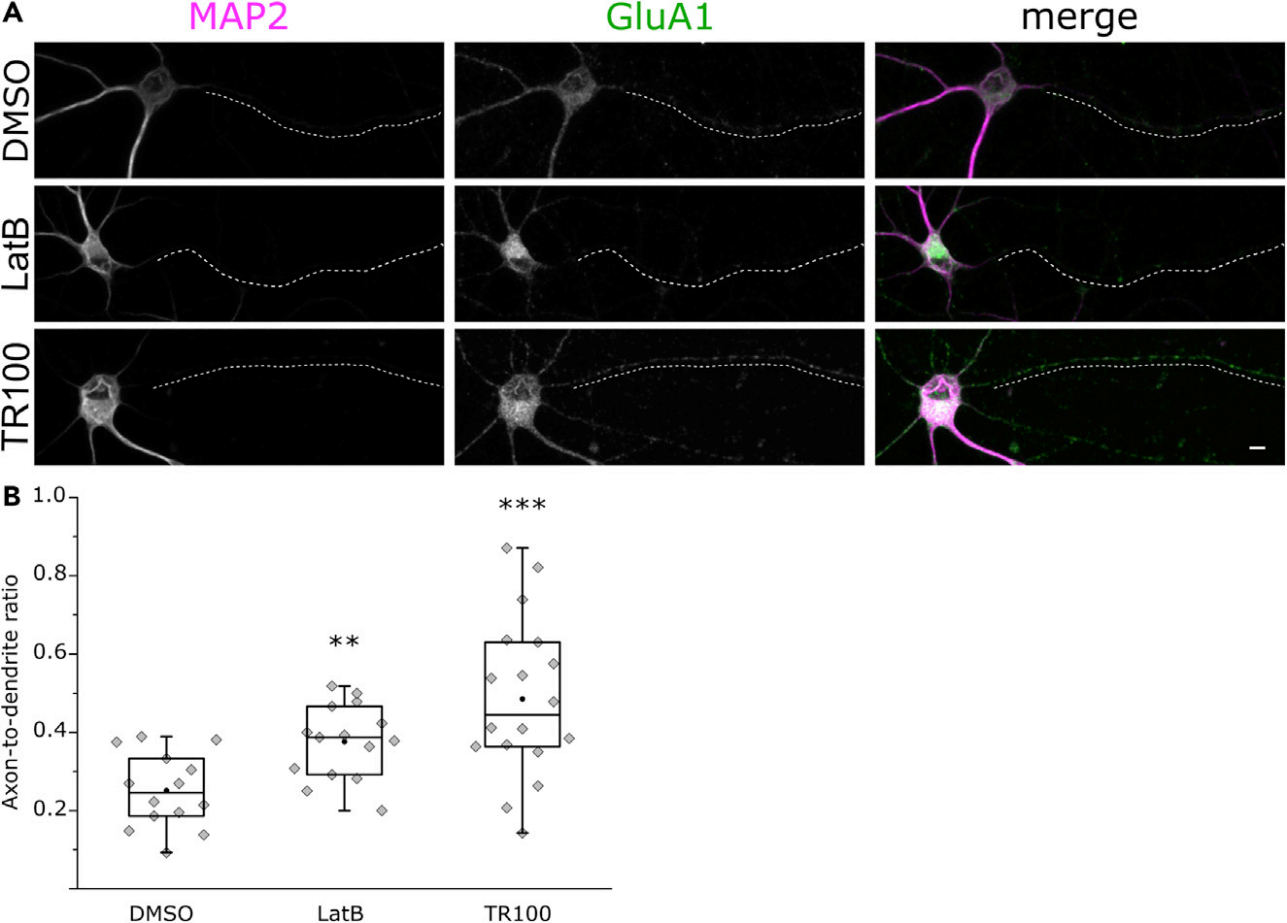
Tpm3.1 Inhibition Leads to the Redistribution of the Somatodendritic Marker GluA1 (A) MAP2 and GluA1 immunofluorescence in rat hippocampal neurons incubated overnight at 9–11 DIV in DMSO, LatB, or TR100. Dashed lines represent axons. (B) GluA1 axon-to-dendrite ratios were higher in LatB- and TR100-treated neurons (Mann-Whitney U test). Black circles represent mean value. Box borders represent the 25^th^ and 75^th^ percentiles, whiskers represent minimum and maximum values less than 1.5x the interquartile range lower or higher than the 25^th^ or 75^th^ percentiles, respectively (Tukey style). DMSO 0.2%: n = 14, 2 independent experiments; LatB 5 μM: n = 15, 2 independent experiments; TR100 5 μM: n = 18, 2 independent experiments. ∗ denotes statistical significance. ∗∗: p < 0.01; ∗∗∗: p < 0.001. Scale bar: 5 μm.

Cargo transport filtering and the diffusion barrier at the AIS require an intact actin cytoskeleton (Song et al., 2009, Winckler et al., 1999). Somatodendritic cargo entering the AIS halt at regions of high F-actin concentration, in a process that is dependent on myosin motors (Balasanyan et al., 2017, Janssen et al., 2017, Watanabe et al., 2012). To test if Tpm3.1 is required for maintaining the AIS cargo transport filter, we fixed sparse cultures of rat hippocampal neurons at 10 DIV after an overnight treatment using DMSO (0.2%), LatB (5 μM), or TR100 (5 μM) and used antibodies against the somatodendritic glutamate receptor subunit GluA1 to visualize its distribution. Anti-MAP2 served to label the somatodendritic domain. Contrary to the somatodendritic localization of GluA1 observed in DMSO-treated neurons, we detected GluA1 immunofluorescence in both the dendrites and axons of treated neurons (Figure 6A). We used maximum intensity projection images from confocal stacks to blindly measure the mean fluorescence intensity along the axon and dendrites to calculate the axon-to-dendrite ratio for each group (Lewis et al., 2009). LatB-treated neurons showed a higher axon-to-dendrite ratio (0.38 ± 0.02, mean ± SEM, n = 15 neurons, 2 independent experiments, p < 0.01, Mann-Whitney U test) than DMSO-treated neurons (0.25 ± 0.03, mean ± SEM, n = 14 neurons, 2 independent experiments). TR100-treated neurons also showed a higher axon-to-dendrite ratio (0.49 ± 0.05, mean ± SEM, n = 18 neurons, 2 independent experiments, p < 0.001, Mann-Whitney U test) (Figure 6B). This suggests that Tpm3.1 function is necessary for maintaining the selectivity of axonal transport at the AIS.

**Figure 7:**
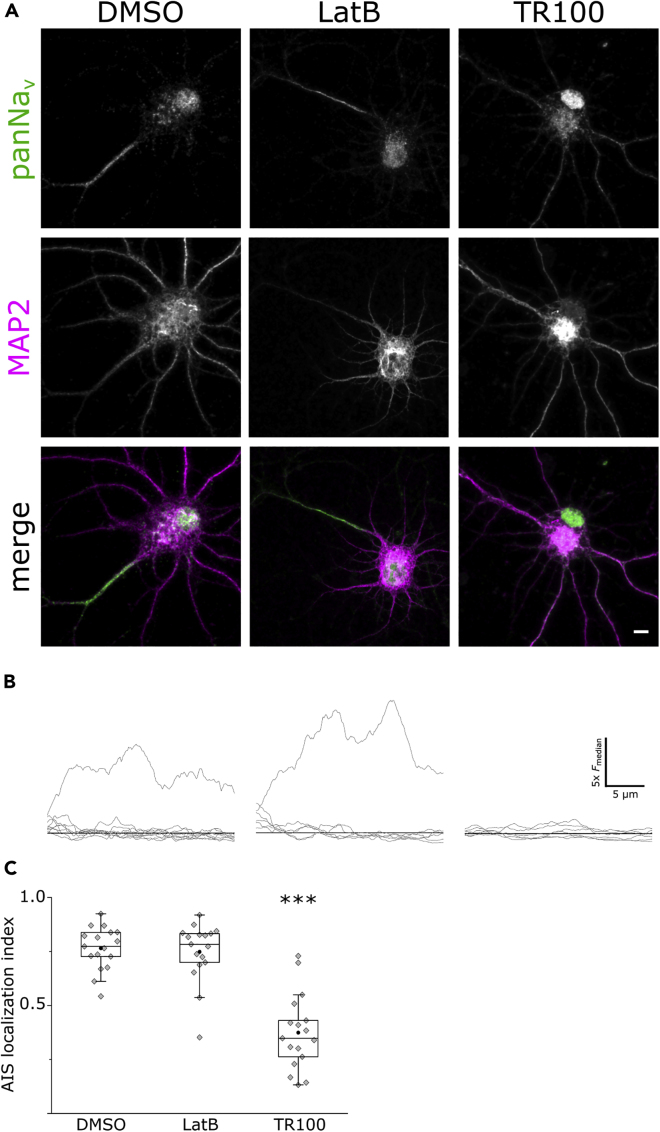
Tpm3.1 Inhibition Leads to the Loss of Voltage-Gated Sodium Channels Clustering at the AIS (A) MAP2 and panNa_v_ immunofluorescence in rat hippocampal neurons incubated overnight at 9–11 DIV in DMSO, LatB, or TR100. (B) Smoothed panNa_v_ fluorescence intensity line profiles (gray lines) along each neurite of the corresponding neuron in (A) normalized to the median value (black line). (C) AIS localization indices for each group (Mann-Whitney U test). Black circles represent mean value. Box borders represent the 25^th^ and 75^th^ percentiles, whiskers represent minimum and maximum values less than 1.5x the interquartile range lower or higher than the 25^th^ or 75^th^ percentiles, respectively (Tukey style). DMSO 0.2%: n = 17, 3 independent experiments; LatB 5 μM: n = 17, 3 independent experiments; TR100 5 μM: n = 17, 3 independent experiments. ∗ denotes statistical significance. ∗∗∗: p < 0.001. Scale bar: 5 μm.

In addition, we wanted to examine the effect of Tpm3.1 inhibition on the clustering of sodium channels, which is essential for spike generation at the AIS (Kole et al., 2008). This accumulation of channels is achieved through interactions with ankyrin G (Jenkins and Bennett, 2001, Zhou et al., 1998). We used antibodies against voltage-gated sodium channels (panNa_v_) and the somatodendritic marker MAP2 to visualize sodium channel clustering at the AIS in sparse cultures of rat hippocampal neurons at 10 DIV after an overnight treatment using DMSO (0.2%), LatB (5 μM), or TR100 (5 μM). DMSO- and LatB-treated neurons showed clear detectable clustering of panNa_v_ immunofluorescence in the AIS, whereas TR100-treated neurons displayed a relatively homogeneous distribution in all neurites (Figure 7A). To quantitatively examine this effect, we used maximum intensity projections of confocal stacks to blindly record panNa_v_ immunofluorescence along the initial 30 μm of each neurite to calculate the ALI (Figure 7B). There was no difference in the mean ALI between DMSO-treated neurons (0.76 ± 0.02, mean ± SEM, n = 17 neurons, 3 independent experiments) and LatB-treated neurons (0.75 ± 0.03, mean ± SEM, n = 17 neurons, 3 independent experiments). In contrast, TR100-treated neurons showed a lower ALI (0.37 ± 0.04, mean ± SEM, n = 17 neurons, 3 independent experiments, p < 0.001, Mann-Whitney U test), indicating a more homogeneous distribution across neurites (Figure 7C). These data suggest that Tpm3.1 is required for the clustering of sodium channels at the AIS.

### Tpm3.1 Inhibition Leads to a Reduction in Firing Frequency and Changes in Action Potential Properties

**Figure 8:**
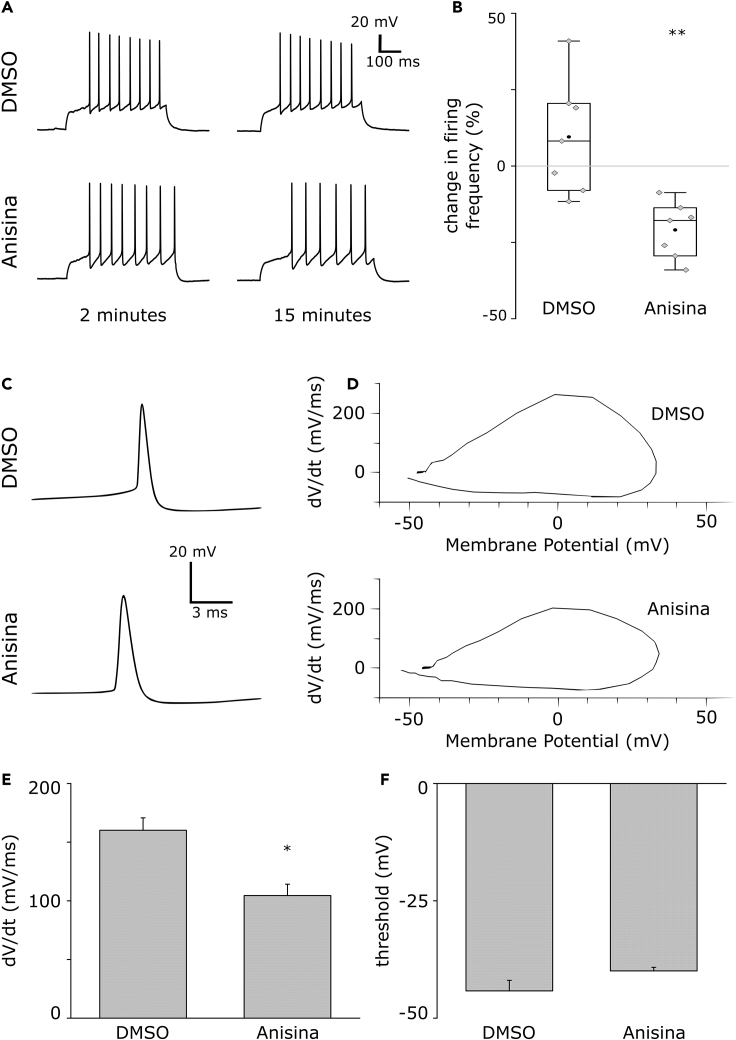
Tpm3.1 Inhibition Leads to a Reduction in Firing Frequency (A) Individual traces from current-clamp (depolarizing step of 100 pA for 500 ms) recordings of rat hippocampal neurons in culture 2 and 15 min after treatments using either DMSO (0.2%) or Anisina (2.5 μM). (B) The percentage change in firing frequency 15 min after introducing each treatment relative to the firing frequency at 2 min. Anisina-mediated inhibition of Tpm3.1 led to the attenuation of firing frequency 15 min after introduction. Black circles represent mean value. Box borders represent the 25^th^ and 75^th^ percentiles, whiskers represent minimum and maximum values less than 1.5x the interquartile range lower or higher than the 25^th^ or 75^th^ percentiles, respectively (Tukey style). DMSO 0.2%: n = 7, 5 independent experiments; Anisina 2.5 μM: n = 7, 5 independent experiments. ∗ denotes statistical significance, two-sample t test. ∗∗: p < 0.01. (C) Representative somatic membrane potential record of an action potential in a cultured hippocampal neuron. The action potential was elicited by current injection of 100 pA. Below, representative somatic membrane potential recording from a cultured hippocampal neuron with the Tpm3.1 inhibitor Anisina (2.5 μM) in the pipette filling solution. (D) Phase plots, the first derivative of the somatic membrane voltage (dV/dt) versus membrane voltage (Vm) for control (DMSO, 0.2%) and Anisina-treated cultured hippocampal neurons. (E) Summary of phase plot slopes 20 mV above threshold. Anisina-treated neurons show a shallower phase plot slope 104.50 ± 9.66 dV/dt compared with control neurons 160.15 ± 10.60, n = 5. ∗ denotes statistical significance, two-sample t test. ∗: p < 0.05. (F) Summary of action potential thresholds at 10 mV/ms; −44.19 ± 2.256 mV for control and −39.93 ± 0.77, p = 0.065 (two-sample t test), n = 5 for Anisina-treated cells, respectively.

The initiation of action potentials is facilitated at the AIS by the clustering of ion channels (Kole et al., 2008) and is dependent on an intact AIS structure (Leterrier et al., 2017). To examine the effect of Tpm3.1 inhibition on spike generation, we recorded the activity of cultured rat hippocampal neurons at 16–18 DIV in current-clamp experiments in the presence of either DMSO (0.2%) or Anisina (2.5 μM). We introduced depolarizing steps of 100–200 pA at 10-s intervals and monitored the firing frequency 2 and 15 min after the introduction of DMSO or Anisina (Figure 8A). The mean firing frequency of DMSO-treated neurons remained unchanged 15 min after the introduction of DMSO (at 2 min: 18.3Hz ± 2.9, mean ± SEM; at 15 min: 20.9Hz ± 4.2, mean ± SEM, n = 7 neurons, 5 independent experiments, paired-sample t test). Conversely, Anisina-treated neurons showed a significant attenuation of firing frequency after 15 min (at 2 min: 21.7Hz ± 4.2, mean ± SEM; at 15 min: 16.7Hz ± 2.6, mean ± SEM, n = 7 neurons, 5 independent experiments, p < 0.05, paired-sample t test). The change in mean firing frequency 15 min after introducing DMSO (9.5% ± 7.0, mean ± SEM) was significantly different from that of neurons treated using Anisina (−20.9% ± 3.4, mean ± SEM, p < 0.01, two-sample t test, Figure 8B).

To study the effects of Tpm3.1 inhibition by Anisina on action potential properties, we constructed phase plane plots using Clampex software (Figure 8D). Tpm3.1 inhibition resulted in a shallower phase plot slope 104.50 ± 9.66 dV/dt compared with control neurons 160.15 ± 10.60, p < 0.05, n = 5 (Figure 8E). The action potential threshold was slightly but not significantly lower in Anisina-treated neurons (−44.19 ± 2.256 and −39.93 ± 0.77 mV for control and Anisina-treated neurons, respectively, p = 0.065, n = 5) (Figure 8F). These data indicate that Tpm3.1 is required for maintaining AIS function in the initiation of action potentials, consistent with the loss of sodium channel clustering at the AIS.

### Tpm3.1 Inhibition Leads to the Gradual Reduction in the Number of Actin Patches and Uniformity of Periodicity of the Sub-membranous Actin Rings

Our results show that Tpm3.1 is important for the accumulation of ankyrin G at the AIS, but it is not clear how Tpm3.1 inhibition leads to the loss of ankyrin G accumulation. We are not aware of any reports suggesting direct interaction between Tpm3.1 and ankyrin G. Thus, we hypothesized that the loss of Tpm3.1 function adversely affects the overall structure and organization of the actin cytoskeleton in the AIS. This disorganization would then ultimately lead to the loss of accumulation of ankyrin G and other structural AIS proteins (Hedstrom et al., 2008, Jenkins and Bennett, 2001, Zhou et al., 1998).

First, we tested the effect of Tpm3.1 inhibition on actin patches in the AIS. Tpm3.1 inhibition led to a reduction in the frequency of actin patches in the AIS compared with DMSO-treated neurons (DMSO: 0.58 ± 0.06 patches/μm, mean ± SEM, n = 13 neurons, 3 independent experiments; LatB: 0.71 ± 0.04 patches/μm, mean ± SEM, n = 13 neurons, 3 independent experiments, p = 0.19; TR100: 0.42 ± 0.02 patches/μm, mean ± SEM, n = 13 neurons, 3 independent experiments, p < 0.05; Anisina: 0.3 ± 0.05 patches/μm, mean ± SEM, n = 12 neurons, 3 independent experiments, p < 0.001, ANOVA, Tukey's test).

**Figure 9:**
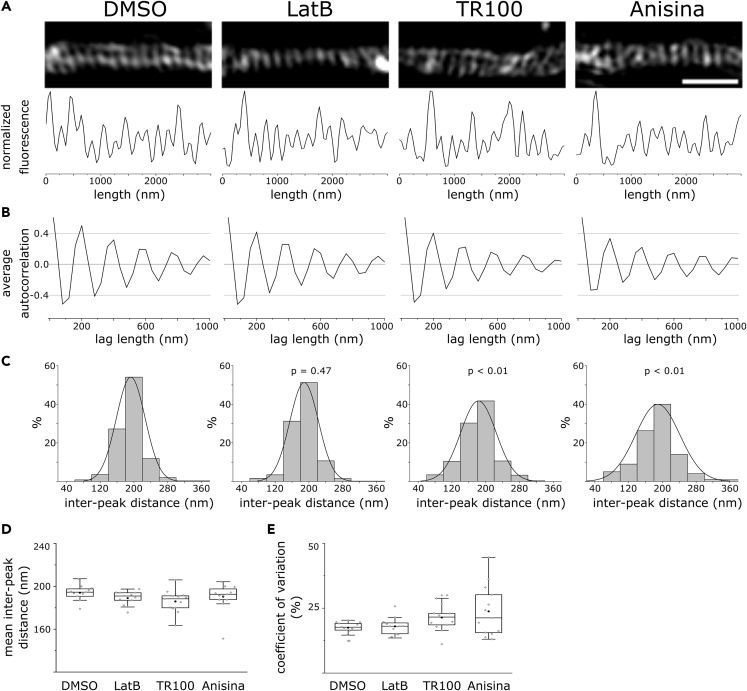
Tpm3.1 Inhibition Disrupts the Periodicity of Actin Rings in the AIS (A) SIM reconstructions of F-actin in the AIS of neurons treated at 14 DIV using DMSO, LatB, TR100, or Anisina (ATM3507), visualized using Alexa 488-tagged phalloidin. (B) Average autocorrelation of normalized fluorescence intensity profiles showing autocorrelation at 200 nm for all groups. (C) Distribution of distances between individual peaks in fluorescence intensity profiles for each group. The distribution of inter-peak distances in TR100- and Anisina-treated neurons was significantly different (p < 0.01) from DMSO- and LatB-treated neurons (Kolmogorov-Smirnov test). (D) The mean inter-peak distance and **e** coefficient of variation for individual cells in each group were not significantly different (Kruskal-Wallis ANOVA, p = 0.05 and p = 0.09, respectively). Black circles (D and E) represent mean value. Box borders represent the 25^th^ and 75^th^ percentiles, whiskers represent minimum and maximum values less than 1.5x the interquartile range lower or higher than the 25^th^ or 75^th^ percentiles, respectively (Tukey style). DMSO: n = 13 neurons, 4 independent experiments; LatB: n = 13 neurons, 4 independent experiments; TR100: n = 13 neurons, 3 independent experiments; Anisina: n = 13 neurons, 3 independent experiments. p values in (C) are relative to DMSO (Kolmogorov Smirnov test). Scale bar: 1 μm. See also Figure S10.

Next, we tested whether Tpm3.1 inhibition affects actin rings. We employed super-resolution microscopy techniques to examine the periodicity of F-actin in the AIS. We treated sparse cultures of rat hippocampal neurons at 14 DIV using DMSO (0.2%), TR100 (10 μM), or Anisina (5 μM) for 6 h. In addition, we treated cultures at DIV 13 using LatB (5 μM) overnight, similar to Winckler et al. (1999). Consistent with our earlier report (Abouelezz et al., 2019), LatB-treated neurons showed an overall lower phalloidin fluorescence intensity, reflecting a decrease in overall F-actin. Periodic actin rings were visible for all groups, indicating the persistence of the sub-membranous lattice, even in the absence of ankyrin G (Figure 9A). We blindly plotted fluorescence intensity profiles in regions within the AIS where periodicity was visible and calculated the autocorrelation function. All groups showed autocorrelation at a lag of 200 nm (Figure 9B). Owing to the pixel size of the camera used (40 nm), the distances recorded are multiples of 40. Accordingly, a lag of 200 nm corresponds to the ∼190 nm reported earlier for actin rings and other components of the AIS sub-membranous lattice (D'Este et al., 2015, Leite et al., 2016, Leterrier et al., 2015, Xu et al., 2013, Zhong et al., 2014). In addition, we blindly measured the distance between individual peaks in each fluorescence intensity profile and compared the distribution of the inter-peak distances across groups (Figure 9C). About 54% of the inter-peak distances in DMSO-treated neurons were 200 nm, whereas the mean inter-peak distance was 192.49 ± 1.37 nm, mean ± SEM. The distribution of the inter-peak distances in LatB-treated neurons was not significantly different from DMSO controls, with 51.2% of the inter-peak distances at 200 nm and a mean inter-peak distance of 188.7 ± 1.38 nm, mean ± SEM (p = 0.47, Kolmogorov Smirnov test). In contrast, TR100-treated neurons showed a less uniform distribution with only 41.7% of the inter-peak distance at 200 nm and a mean inter-peak distance of 182.7 ± 1.74 nm, mean ± SEM (p < 0.01, Kolmogorov Smirnov test). Similarly, only 39.8% of the distances measured in Anisina-treated neurons were 200 nm, with a mean inter-peak distance of 189.0 ± 1.94 nm, mean ± SEM, a distribution significantly different from that of DMSO controls (p < 0.01, Kolmogorov Smirnov test). We also obtained similar results using stochastic optical reconstruction microscopy (STORM) (Figure S10). When calculated on a cell-by-cell basis, the mean inter-peak distance in each group was not significantly different (Kruskal-Wallis ANOVA, p = 0.05). The mean coefficient of variation was also not significantly different among groups (Kruskal-Wallis ANOVA, p = 0.09).

These results show that Tpm3.1 inhibition affects both AIS actin patches and actin rings. Tpm3.1 inhibition reduced the number of actin patches in the AIS, whereas LatB treatment increased their number. As shown before (Abouelezz et al., 2019), we found that the periodicity of sub-membranous actin rings in the AIS was resistant to LatB. As LatB disrupts the actin cytoskeleton through sequestering free actin monomers (thus inhibiting actin polymerization), stable actin filaments with a low rate of depolymerization may be less susceptible to LatB. In contrast, the inhibition of Tpm3.1 for the duration of the experiments (6 h) disrupted—but did not entirely abolish—the periodicity of sub-membranous actin rings. In addition to changing the uniformity of the periodicity, visual inspection revealed that actin rings were often tilted after Tpm3.1 inhibition, losing their nature of parallel transverse stripes (Figure 9A).

### Tpm3.1 Depletion Reduces Myosin IIB Expression

**Figure 10:**
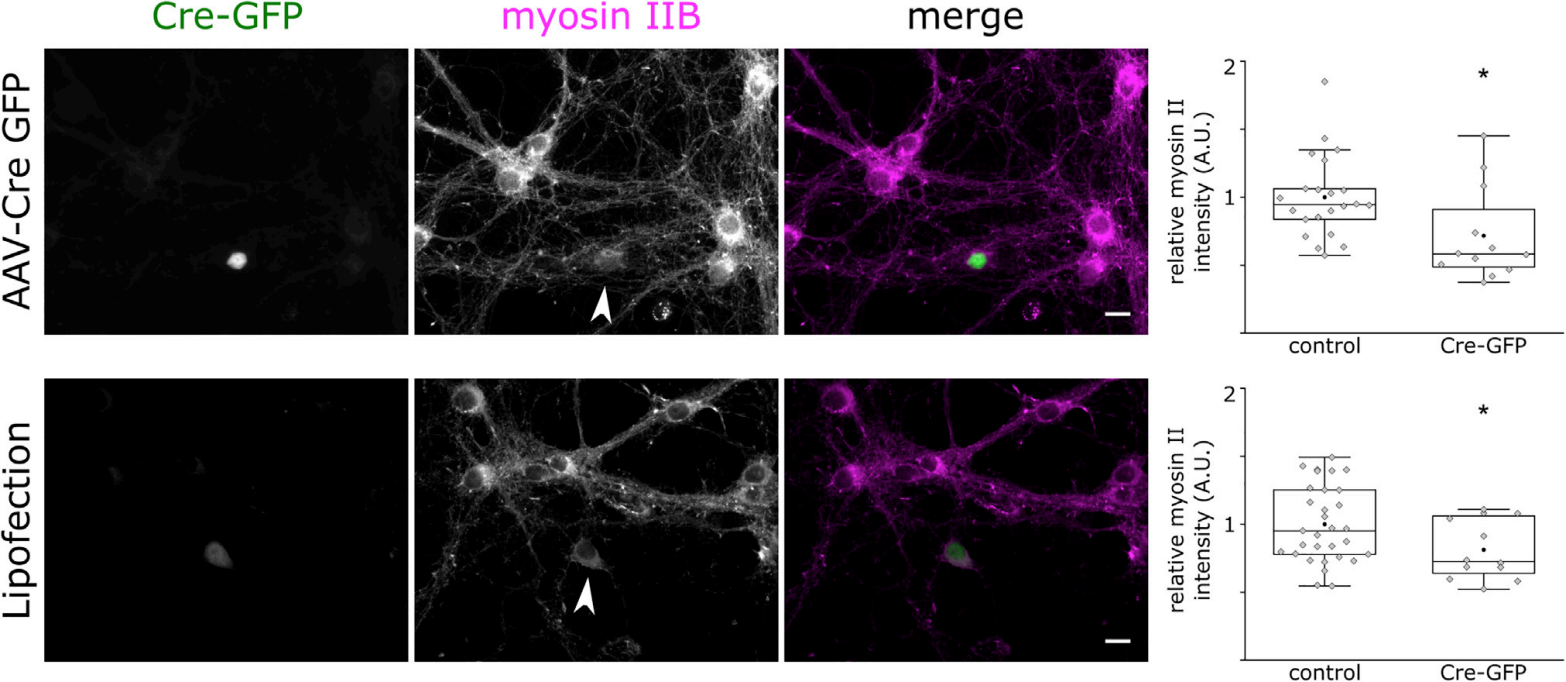
Loss of Tpm3.1 Leads to a Reduction in Myosin IIB Immunofluorescence Cultured hippocampal neurons of conditional *Tpm3* knockout mice (Tp9 line). Arrowheads indicate neurons expressing Cre-GFP after either viral transduction (top panel) or lipofection (bottom panel). We used anti-myosin IIB to compare the distribution of myosin IIB in neurons expressing Cre-GFP and neighboring control neurons. Cre-GFP expressing neurons showed a lower intensity of myosin IIB immunofluorescence (t test). Box borders represent the 25^th^ and 75^th^ percentiles, whiskers represent minimum and maximum values less than 1.5x the interquartile range lower or higher than the 25^th^ or 75^th^ percentiles, respectively (Tukey style). Neurons expressing Cre-GFP; transduced: n = 12, 3 independent experiment; transfected n = 12, 3 independent experiments. Control neurons: transduced: n = 22; transfected: n = 31. ∗ denotes statistical significance, two-sample t test. ∗: p < 0.05. Scale bar: 10 μm.

The earlier experiments have mainly followed the idea that Tpm3.1 affects actin filament stability. However, in addition to playing a role in F-actin turnover, Tpm3.1 recruits and activates myosin IIB (Bryce et al., 2003, Gateva et al., 2017), and recent work has revealed an important role for myosin II in AIS structure (Berger et al., 2018, Evans et al., 2017, Wang et al., 2020). In U2 Osteosarcoma cells, Tpm3.1/3.2 overlaps with non-muscle myosin II heads but not with non-muscle myosin II tails or α-actinin in stress fibers (Meiring et al., 2019). Thus, we examined the effect of perturbing Tpm3.1 in cultured neurons on myosin IIB. We expressed Cre-GFP in cultured hippocampal neurons of *Tpm3* conditional knockout mice (Tp9 line) using either viral transduction or lipofection and used anti-myosin IIB to examine myosin IIB distribution (Figure 10). Neurons expressing Cre-GFP after viral transduction showed a lower intensity of myosin IIB immunofluorescence (0.72 ± 0.1, mean ± SEM, n = 12) relative to neighboring control neurons (1 ± 0.06, mean ± SEM, p < 0.05, two-sample t test, n = 22, 3 independent experiments). Similarly, compared with neighboring control neurons (1 ± 0.05, mean ± SEM, n = 31), neurons expressing Cre-GFP after lipofection showed a lower intensity of myosin IIB immunofluorescence (0.81 ± 0.06, mean ± SEM, p < 0.05, two-sample t test, n = 12, 3 independent experiments). The results are summarized in Figure 10.

## Discussion

Although an intact actin cytoskeleton is required for the formation of the AIS (Xu and Shrager, 2005), the mature AIS is remarkably stable and insensitive to actin-disrupting drugs (Jones et al., 2014, Leterrier et al., 2015, Sanchez-Ponce et al., 2011, Song et al., 2009). This may lead to the conclusion that the actin cytoskeleton has no significant role in the maintenance of AIS structure. The loss of accumulation of ankyrin G (Figures 4, 5, S5, and S9) and other AIS structural (Figure S5) and functional proteins (Figure 7), disruption in sorting somatodendritic and axonal proteins (Figure 6), and a reduction in firing frequency (Figure 8) upon the perturbation of Tpm3.1 function, however, suggest otherwise.

The AIS actin cytoskeleton comprises sub-membranous actin rings and actin-rich patches (Papandreou and Leterrier, 2018). Although sub-membranous actin rings are not exclusive to the AIS (Bertling and Hotulainen, 2017, D'Este et al., 2015, Han et al., 2017, Leite et al., 2016, Leite and Sousa, 2016, Xu et al., 2013, Zhong et al., 2014), actin patches in the AIS are more numerous and resistant to extraction, compared with more distal axonal patches (Balasanyan et al., 2017). Our data suggest that Tpm3.1 co-localizes with actin patches in the AIS (Figure 2 and S2) and that the inhibition of Tpm3.1 led to a reduction in the number of AIS actin patches. In addition to patches, Tpm3.1 showed a periodic distribution similar to sub-membranous actin rings but Tpm3.1 was only partially congruent with sub-membranous actin rings (Figures 3 and S4). Nevertheless, the inhibition of Tpm3.1 affected the alignment of actin rings and the uniformity of their periodicity (Figures 9 and S10). In addition to rings and patches, axons have other less characterized actin structures such as actin trails or hotspots (Ganguly et al., 2015). As the distribution of Tpm3.1 was not congruent with sub-membranous actin rings (Figure S4), we propose that Tpm3.1 decorated actin filaments are partially actin filaments of actin rings but partially actin filaments outside the rings. Currently it is unclear what exactly are the Tpm3.1 decorated filaments outside the rings and patches. It is also possible that the inhibition of Tpm3.1 affects the recruitment and accumulation of other actin-binding proteins that are important for maintaining actin rings or other actin structures in the AIS. Although the exact mechanism is still unclear, our data show that Tpm3.1 provides support for the AIS structural complex.

### Tpm3.1 Stabilizes Actin Filaments and Recruits and Activates Myosin II

Biochemically, Tpm3.1 was shown to have two main functions in actin filament regulation—it stabilizes actin filaments and regulates myosin IIB binding and contractility. Tpm3.1 enhances the phosphorylation (and inactivation) of actin-depolymerizing factor/cofilin (Bryce et al., 2003) and inhibits the binding of cofilin to the pointed ends of F-actin (Jansen and Goode, 2019), thus inhibiting filament severing as well as depolymerization at the pointed ends (Broschat, 1990). Furthermore, Tpm3.1 recruits tropomodulin to the pointed ends (Sung and Lin, 1994), further lowering the rate of depolymerization (Weber et al., 1994, Yamashiro et al., 2014). Thus, the inhibition of Tpm3.1 renders Tpm3.1-decorated actin filaments vulnerable to depolymerization (Bonello et al., 2016), and therefore, it is plausible that the loss of AIS structure upon Tpm3.1 inhibition is due to a reduction in the stability of actin filaments. Bach et al. (2009) showed that Tpm3.1 is required for stabilizing actin filaments in the formation and maturation of focal adhesions. Tpm3.1-decorated actin filaments are the least sensitive to latrunculin and Cytochalasin D (Creed et al., 2008, Percival et al., 2000). It is, therefore, expected that inhibiting Tpm3.1 function will have a substantial effect on actin filament dynamics in the AIS. Altering the stability, length, or linearity of the actin filaments building the AIS may then lead to less organized structures (Tojkander et al., 2011).

In addition to actin filament stabilization, Tpm3.1 recruits and activates myosin II (Bryce et al., 2003, Gateva et al., 2017, Meiring et al., 2019), which has recently emerged as an important regulator of AIS structure (Berger et al., 2018, Evans et al., 2017). Recent super-resolution and electron microscopy studies reported a subcellular localization of myosin IIB similar to what we detected for Tpm3.1 (Vassilopoulos et al., 2019, Wang et al., 2020). Myosin II also shows periodicity but not as uniform as actin, and it only partially overlaps with actin rings. Immunogold labeling for pMLC followed by PREM showed gold beads often appearing along filaments perpendicular to actin braids (Vassilopoulos et al., 2019). It is thus plausible that Tpm3.1 further contributes to the structure of the AIS by recruiting myosin II to the fibrillar coat, providing the lattice with contractile characteristics. This is supported by the relatively low myosin IIB immunofluorescence in *Tpm3* KO neurons (Figure 10). However, the reduced myosin IIB expression in Tpm3.1 KO cells is just the first indication of possible interaction of Tpm3.1 and myosin IIB in neurons. Further experiments are required to clarify how Tpm3.1 affects myosin IIB in the AIS. One more alternative explanation for the loss of AIS structure upon Tpm3.1 inhibition could be that Tpm3.1 depletion or inhibition perturbs the specific interactions between Tpm3.1 and proteins contributing to the structure of the AIS; for example, by regulating the binding of βIV-spectrin to the sub-membranous actin rings. Again, further experiments are needed to elucidate which scenario is the correct one.

### Tpm3.1 Inhibitors and Genetic Manipulations Affect Actin Filaments via Different Mechanisms

We used here three techniques (small molecule inhibitors, conditional knockout, and shRNA) to perturb Tpm3.1 function or expression. Although the results all point in the same direction, Tpm3.1 inhibitors resulted in a stronger decrease in ankyrin G intensity compared with genetic manipulation. However, there are good reasons for this difference. First, genetic manipulation (conditional knockout and shRNA) decreased Tpm3.1/2 levels but with limited efficacy. This already gives an understandable explanation for the milder effects when protein levels are partially decreased versus an approach where protein function is totally blocked. Furthermore, genetic perturbation and drugs affect filaments differently. Genetic depletion leads to longer-lasting protein level decrease, and loss of a specific filament population can lead to compensating changes. In contrast, the anti-Tpm3.1 drugs do not prevent assembly of Tpm3.1-containing actin filaments (Bonello et al., 2016, Janco et al., 2019). Rather, the drugs incorporate into the Tpm3.1/actin filament and alter the function and stability of the filament (Bonello et al., 2016, Currier et al., 2017, Janco et al., 2019). In addition, their effects are quick, which avoids the likelihood of compensatory changes in the cytoskeleton. Thus, even with 100% knockout efficiency, the drugs are expected to give more penetrant phenotypes than that observed with knockout and shRNA strategies. It is also important to note that, for the main result, we used two structurally distinct Tpm3.1 inhibitors, TR100 and Anisina, and hence, it is highly unlikely to have the same off-target impact.

### Tpm3.1 Inhibition Perturbs the Functionality of the AIS

It has been suggested that actin patches are important for cargo transport filtering (Janssen et al., 2017, Leterrier and Dargent, 2014, Watanabe et al., 2012). Tpm3.1 inhibition indeed reduced the number of actin patches in AIS. In contrast, LatB treatment did not decrease the number of patches in the AIS, although it made the AIS leaky. It is possible, however, that LatB affected the turnover and functionality of the actin filaments in patches. Thus, the exact mechanism of how the actin cytoskeleton contributes to the regulation of cargo transport filtering is still unclear.

The effect of Tpm3.1 inhibition by Anisina on firing frequency was relatively rapid (Figure 8) suggesting that the high-order structure of the AIS, which is required for proper functionality of voltage-gated sodium channels is highly sensitive to Tpm3.1 inhibition. Phase-plane plots revealed shallower slopes of action potentials at +20 mV from threshold in Tpm3.1 blocked neurons, compared with control (Figures 8C–8F), thus suggesting more proximal action potential initiation sites (Kress et al., 2008). The result is consistent with our immunofluorescence data showing redistribution and clustering of voltage-gated sodium channels to more somatic location in Tpm3.1-blocked neurons (Figure 7). The finding is also in line with Kress et al. (2008) who showed that a shallower action potential slope is associated with more proximal voltage-gated sodium channel clustering in hippocampal granule cell fibers in comparison with CA3 pyramidal cells having a steeper action potential slope and more distal sodium channel clustering. However, since 15 min Anisina treatment may not have been sufficient to induce marked structural rearrangement of sodium channel localization within the axon, other mechanisms decreasing axonal voltage-gated sodium channel function, such as disruption of sodium channels' order or density at the AIS, may have come to play here. Taken together, Anisina treatment decreases excitability of neurons in a manner consistent with reduced functional sodium channel density at the AIS. Although the mechanistic details await further study, the electrophysiological data give strong functional support to our findings on the redistribution/functional change of sodium channels after Tpm3.1 inhibition.

Taken together, we showed a novel characterization of Tpm3.1 as an AIS component and that its expression and functionality are required for the normal maintenance and functionality of AIS in primary hippocampal neurons.

## Limitations of the Study

This study was limited to cultured primary hippocampal neurons. In the future, results need to be confirmed in *in vivo* models. It will also be interesting to see whether Tpm3.1 functionality is needed for other cell types. Based on our results, we expect that Tpm3.1-facilitated organization of the AIS is not needed in some neuron types, such as granule cells, where sodium channels are clustered at a different location. Furthermore, for example, Neurofascin depletion has been shown to have different influences in Purkinje cells versus hippocampal neurons (Zonta et al., 2011, Leterrier et al., 2017). Currently, it is unclear whether different neuron types have different organizations and protein compositions in the AIS, but this is a very interesting study question for the future.

The Tpm3.1 isoform, as most tropomyosin isoforms, is nearly impossible to study without affecting or detecting other tropomyosin isoforms. In this study, conditional knockout was depleting all *Tpm3* gene products and our shRNA was depleting Tpm3.1 and its close homologue Tpm3.2. Antibodies used against Tpm3.1 recognize both Tpm3.1 and Tpm3.2. Genetic depletion of Tpm3.1/2, both with conditional knockout cells (where depletion was induced with Cre infection) and shRNA-induced depletion, was limited. It may indicate that the degradation turnover of Tpm3.1/2 is relatively slow.

## Methods

All methods can be found in the accompanying Transparent Methods supplemental file.

## Data and Code Availability

Original data have been deposited to Mendeley Data: https://doi.org/10.17632/jw64nf3rn5.1.

## Supplemental Information

Document S1. Transparent Methods and Figures S1–S10
