# Supplementary material for: Tropomyosin Tpm3.1 Is Required to Maintain the Structure and Function of the Axon Initial Segment
Source: iScience. 2020 Apr 12;23(5):101053. doi: 10.1016/j.isci.2020.101053 (PMC7186529; doi:10.1016/j.isci.2020.101053)
Supplement: Document S1. Transparent Methods and Figures S1–S10 [file mmc1.pdf]

iScience, Volume *23*

Supplemental Information

Tropomyosin Tpm3.1 Is Required to Maintain the Structure and Function of the Axon Initial Segment

Amr Abouelezz, Holly Stefen, Mikael Segerstråle, David Micinski, Rimante Minkeviciene, Lauri Lahti, Edna C. Hardeman, Peter W. Gunning, Casper C. Hoogenraad, Tomi Taira, Thomas Fath, and Pirta Hotulainen

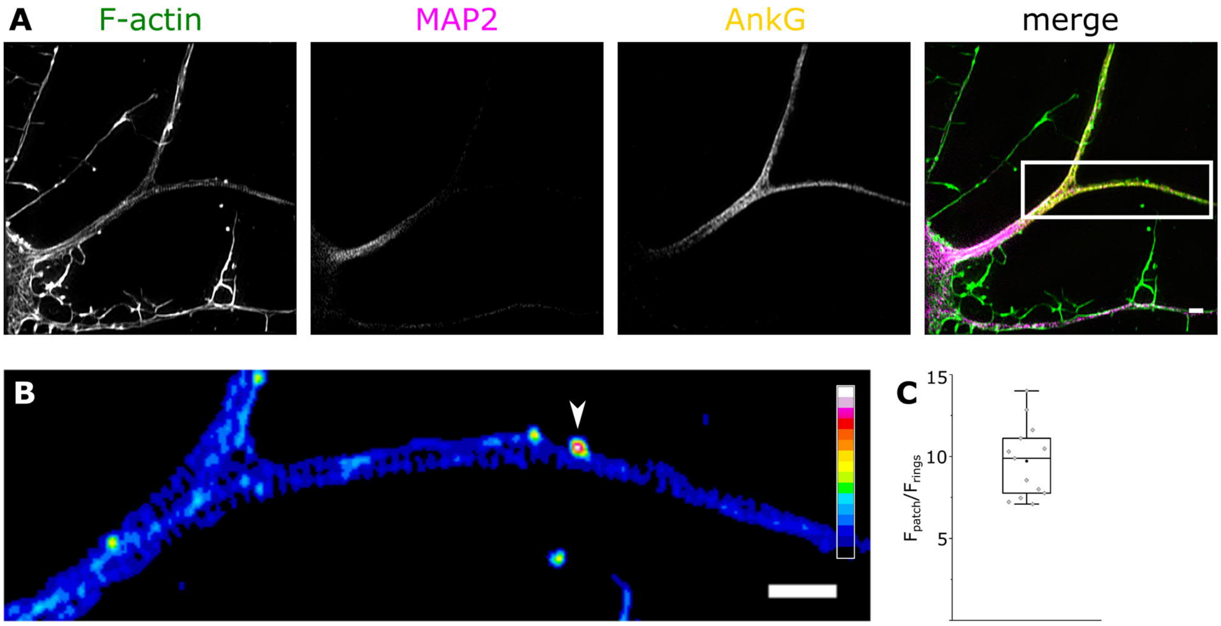

Supplementary figure 1 (related to Figure 1) | AIS actin patches contain more F-actin than sub-membranous actin rings.

(A) SIM reconstructions of rat hippocampal neuron at 14 DIV stained using Alexa 488-tagged phalloidin, anti-MAP2, and anti-ankyrin G. The boxed region is enlarged in (B).

(B) Maximum intensity projection of a SIM reconstruction of F-actin in the boxed region, corresponding to the AIS. Color code indicates normalized fluorescence intensity levels. Arrowhead indicates F-actin patch.

(C) The median fluorescence intensity of phalloidin in AIS actin patches was, on average, 9.7 times that of sub-membranous actin rings. Black circle represents mean value. Box borders represent the 25th and 75th percentiles, whiskers represent minimum and maximum values less than 1.5x the interquartile range lower or higher than the 25th or 75th percentiles, respectively (Tukey style). n = 13, 4 independent experiments, same data set used in Fig. 9. Scale bar: 1 µm.

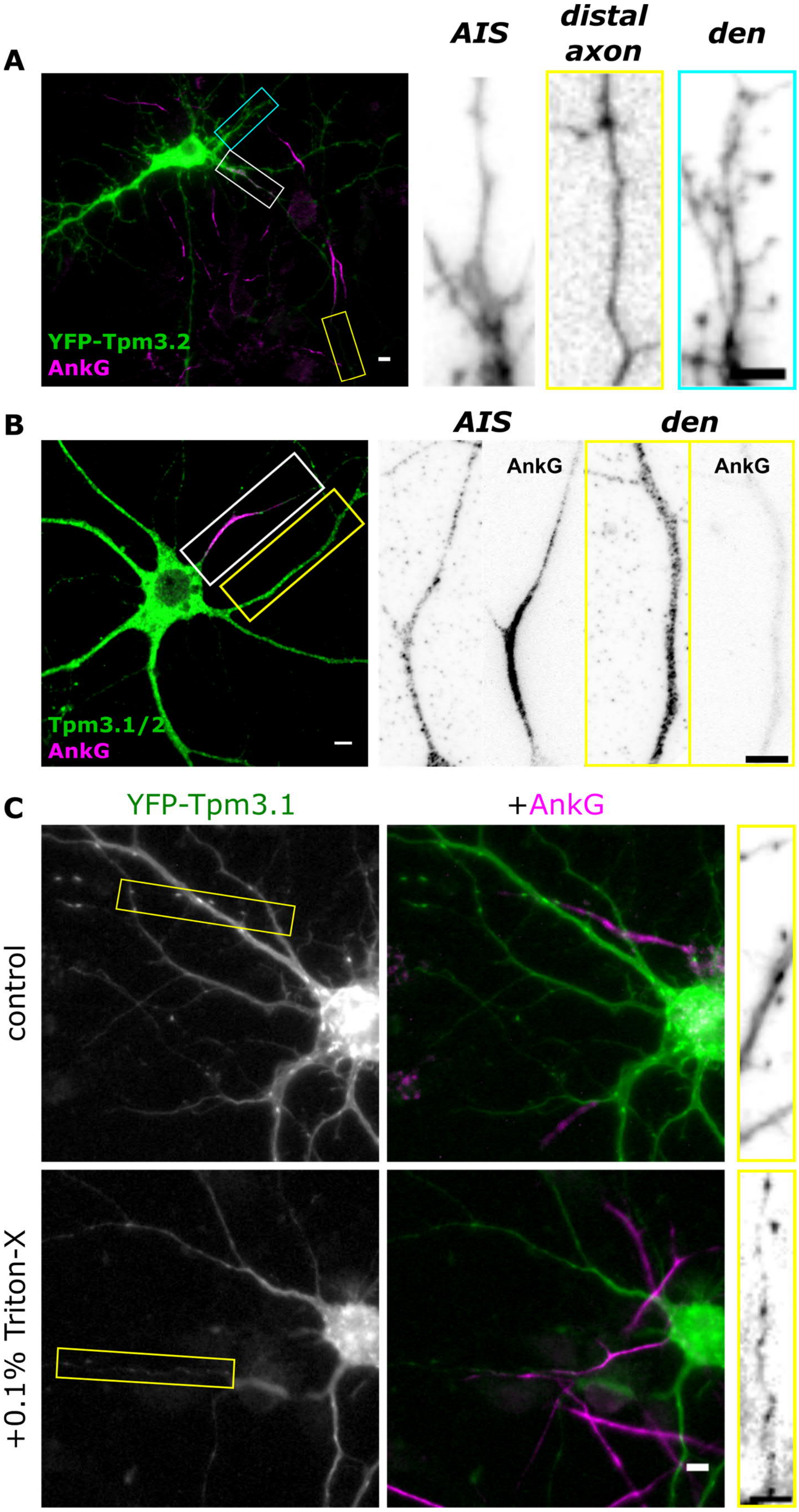
A
AIS
distal axon
den
YFP-Tpm3.2
AnkG
B
AIS
den
AnkG
AnkG
Tpm3.1/2
AnkG
C
YFP-Tpm3.1
+AnkG
control
+0.1% Triton-X

Supplementary figure 2 (related to Figure 2) | Tpm3.1 shows a non-uniform distribution similar to AIS actin patches.

(A) Rat hippocampal neurons expressing YFP-Tpm3.2. Neurons were fixed 8 hours post-transfection. Anti-ankyrin G served to label the AIS. Compared to YFP-Tpm3.1, YFP-Tpm3.2 shows a diffuse staining in the AIS (white box) and distal regions of the axon (yellow box), and a much stronger presence in the somatodendritic compartment (cyan box) and dendritic spines. Scale bar: 10 µm.

(B) Tpm3.1 was visualized in rat hippocampal neurons at 10 DIV using anti-γ/9d. Anti-ankyrin G served to label the AIS. Anti-γ/9d simultaneously detects Tpm3.1 and Tpm3.2. Anti-γ/9d immunofluorescence in the AIS was unevenly distributed in the AIS, with patches of high intensity similar to AIS actin patches. In contrast, anti-γ/9d in the somatodendritic domain was diffuse and ubiquitous. Scale bar: 5 µm.

(C) Rat hippocampal neurons expressing YFP-Tpm3.1 were either fixed or extracted in 0.1% Triton-X for 60 seconds then fixed 8 hours post-transfection. Anti-ankyrin G served to label the AIS. Patches of YFP-Tpm3.1 can still be seen in the AIS after detergent extraction. Scale bar: 5 µm.

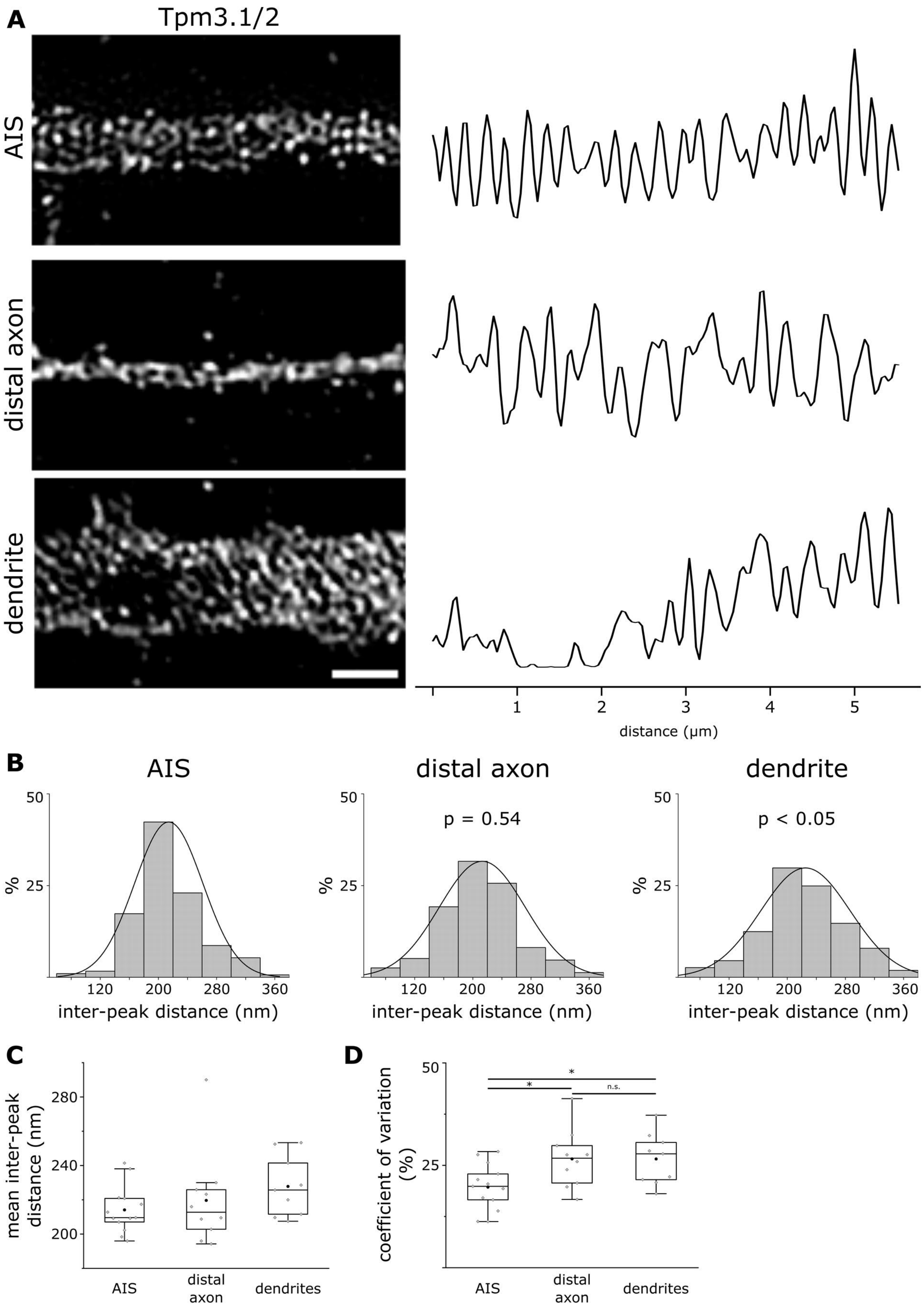
A
Tpm3.1/2
AIS
distal axon
dendrite
1
2
3
4
5
distance (µm)
B
AIS
distal axon
dendrite
50
%
25
120
200
280
360
inter-peak distance (nm)
p = 0.54
p < 0.05
C
mean inter-peak distance (nm)
280
240
200
AIS
distal axon
dendrites
D
coefficient of variation (%)
50
25
*
*
n.s.
AIS
distal axon
dendrites

Supplementary figure 3 (related to Figure 3) | Tpm3.1 does not exhibit clear periodicity in the distal axon or in dendrites of cultured rat hippocampal neurons.

(A) *Left*: SIM reconstructions of the AIS, a distal region of the axon, and a dendrite of a rat hippocampal neuron at 14 DIV stained using anti-γ/9d. Periodic Tpm3.1/2 immunofluorescence is visible in the AIS, but not in the distal regions of the axon or in dendrites. *Right*: Anti-γ/9d fluorescence intensity profiles from the corresponding images. Scale bar: 1 µm.

(B) Distance between individual peaks in normalized Tpm3.1 fluorescence intensity profiles. In the AIS, 42.3% of the peaks were separated by 200 nm. In distal axons and dendrites, the corresponding values were 31.6% and 29.8%, respectively. The inter-peak distances in distal axons were not significantly different compared to the AIS, while in dendrites the distribution was significantly different (Kolmogorov-Smirnov test).

(C and D) The mean inter-peak distance (C) for individual cells at the AIS, distal axon, or dendrites was not significantly different (ANOVA), while the coefficients of variation (D) were higher in the distal axon and dendrites compared to the AIS ($p < 0.05$, ANOVA, Tukey's test). Box borders represent the 25th and 75th percentiles, whiskers represent minimum and maximum values less than 1.5x the interquartile range lower or higher than the 25th or 75th percentiles, respectively (Tukey style). * denotes statistical significance. *: $p < 0.05$.

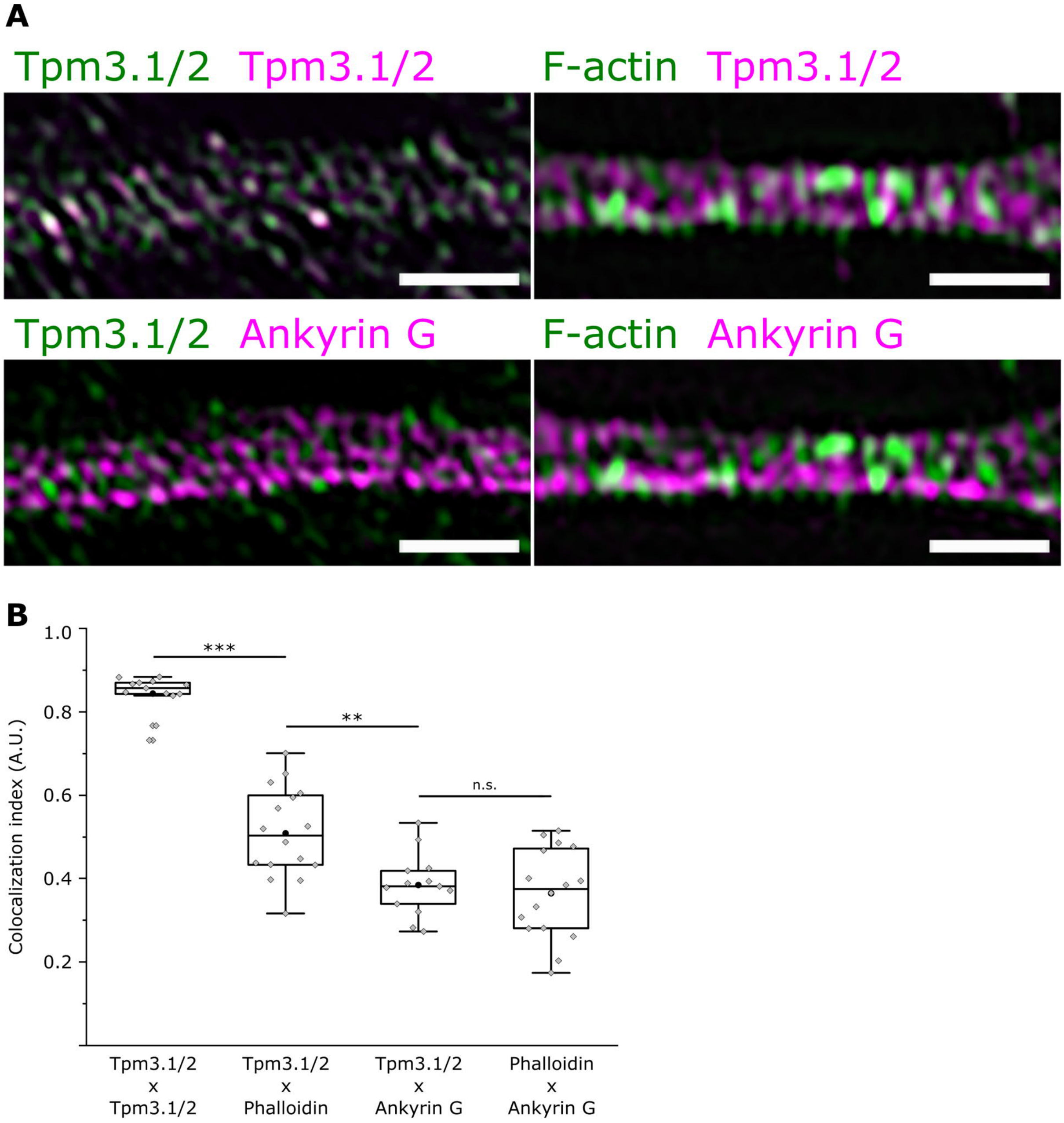

Supplementary figure 4 (related to Figure 3) | F-actin and Tpm3.1 exhibit partial overlap.

(A) SIM reconstruction of the AIS of a rat hippocampal neuron at 14 DIV labelled using anti-γ/9d and phalloidin to visualize Tpm3.1 and F-actin, respectively. *Left:* cells were stained using two different secondary antibodies, Alexa-488 and 647-tagged, to detect anti-γ/9d. *Right:* Cells were stained using Alexa 488-tagged phalloidin and anti-γ/9d followed by an Alexa 647-tagged secondary antibody. Anti-Ankyrin G served to label the AIS. Ankyrin G labeling was used as a negative control (F-actin and ankyrin G show complementary distribution) in co-localization analysis. Scale bar: 1 µm.

(B) The double-labelled Tpm3.1 showed a Pearson's coefficient of colocalization (PCC) of 0.84 ± 0.01 (mean ± SEM), while the negative control (phalloidin x ankyrin G) showed a PCC of 0.36 ± 0.03. Tpm3.1 x ankyrin G showed a similar PCC (0.38 ± 0.02) to the negative control, suggesting that Tpm3.1/2 and ankyrin G have complementary localization. Tpm3.1/2 x phalloidin showed partial co-localization, with a significantly different co-localization index of 0.51 ± 0.03 compared to both positive and negative controls (ANOVA, Tukey's test). Black circle represents mean value. Box borders represent the 25th and 75th percentiles, whiskers represent minimum and maximum values less than 1.5x the interquartile range lower or higher than the 25th or 75th percentiles, respectively (Tukey style). Neurons double-labelled with Tpm3.1/2 and ankyrin G: n = 13, Neurons labeled with phalloidin, ankyrin G and Tpm3.1/2: n = 16. * denotes statistical significance. **: $p < 0.01$; ***: $p < 0.001$.

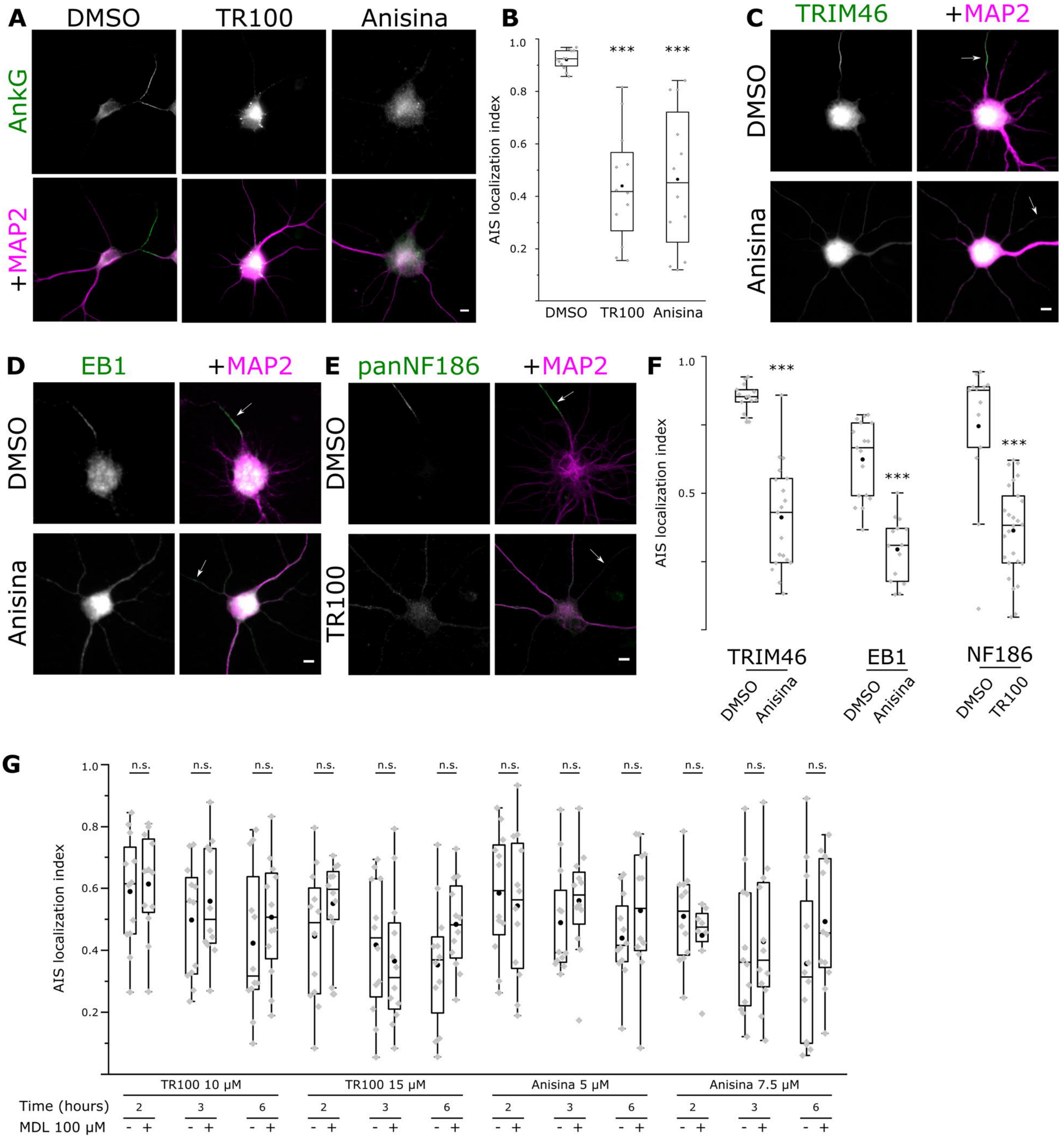

Supplementary figure 5 (related to Figure 4) | Overnight inhibition of Tpm3.1 reduces the accumulation of ankyrin G and other AIS markers at the AIS.

(A) Rat hippocampal neurons treated overnight at 9 DIV using DMSO or the small-molecule Tpm3.1 inhibitors TR100 or Anisina (ATM3507). Anti-MAP2 served to label the somatodendritic domain, anti-ankyrin G served to measure the accumulation of ankyrin G.

(B) AIS localization indices for neurons treated using DMSO, TR100 (5 µM), or Anisina (2.5 µM). Both TR100- (mean ALI: 0.44 ± 0.06, mean ± SEM) and Anisina-treated neurons (mean ALI: 0.48 ± 0.09, mean

± SEM) were significantly different from DMSO controls (mean ALI: 0.92 ± 0.01, mean ± SEM; Mann-Whitney *U* test). Black circles represent mean values. For each treatment: n = 12, 3 independent experiments. Scale bar: 5 µm.

(C, D, and E) Rat hippocampal neurons treated at 9-11 DIV using, Anisina (5 µM for 6 hours), TR100 (5 µM overnight), or equivalent volumes of DMSO. Anti-MAP2 served to label the somatodendritic domain. Anti-TRIM46 (C), anti-EB1 (D), or panNF-186 (E) served to visualize the corresponding AIS marker. DMSO-treated neurons showed typical accumulation of TRIM46 and EB1, which are implicated in the regulation of microtubules, as well as the AIS-specific adhesion protein neurofascin-186 (NF-186). Anisina- or TR100-treated neurons showed no accumulation of any of these proteins at the AIS. Scale bar: 5 µm.

(F) AIS localization indices for anti-TRIM46, anti-EB1- or anti-NF186. The ALI was significantly lower in all Anisina- or TR100-treated neurons: TRIM46; DMSO: 0.84 ± 0.01, mean ± SEM, n = 14, 3 independent experiments, Anisina: 0.41 ± 0.04, mean ± SEM, n = 19, 3 independent experiments, p < 0.001, *t*-test; EB1; DMSO: 0.62 ± 0.03, mean ± SEM, n = 17, 3 independent experiments, Anisina: 0.29 ± 0.03, mean ± SEM, n = 14, 3 independent experiments, p < 0.001, *t*-test; NF-186; DMSO: 0.75 ± 0.07, mean ± SEM, n = 13, 3 independent experiments, TR100: 0.36 ± 0.03, mean ± SEM, n = 26, 3 independent experiments, p < 0.001, *t*-test.

(G) AIS localization indices (ALI) for ankyrin G in rat hippocampal neurons treated at 10 DIV using the small-molecule Tpm3.1 inhibitors TR100 or Anisina (ATM3507) for 2, 3, or 6 hours, in the presence or absence of the calpain inhibitor MDL28170 (100 µM). The presence of MDL28170 did not significantly affect the ALI for any of the treatments (Mann-Whitney *U* test). Anisina 7.5 µM + MDL28170 100 µM, 2 hours: n = 8, 2 independent experiments; all other treatments: n = 12, 3 independent experiments. Part of these data were used for Figure 4. Black circles represent mean value. Box borders represent the 25th and 75th percentiles, whiskers represent minimum and maximum values less than 1.5x the interquartile range lower or higher than the 25th or 75th percentiles, respectively (Tukey style). * denotes significance. ***: p < 0.001; n.s.: not significant.

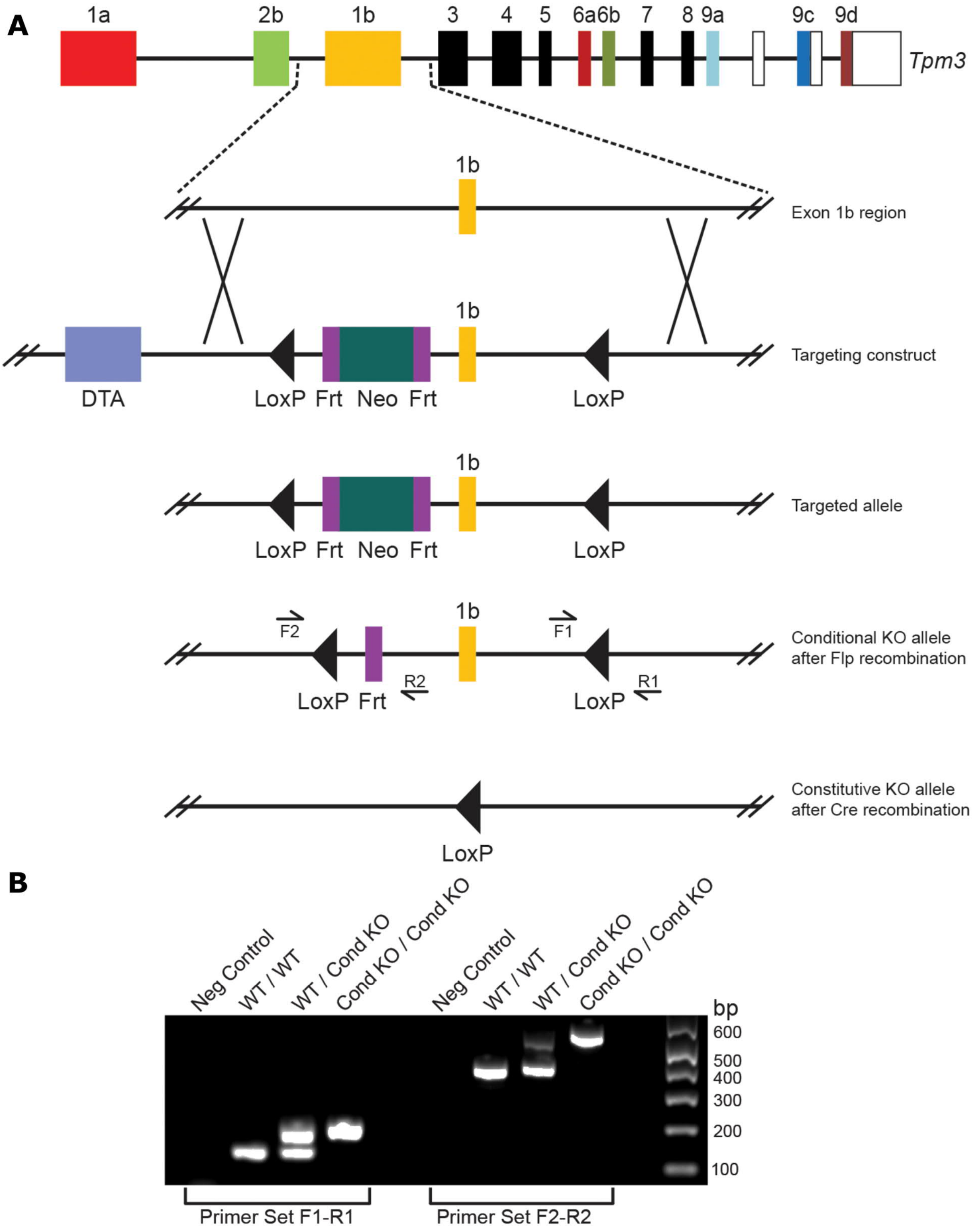

A
1a
2b
1b
3
4
5
6a6b
7
8 9a
9c
9d
Tpm3
1b
Exon 1b region
1b
DTA
LoxP Frt Neo Frt
LoxP
Targeting construct
1b
LoxP Frt Neo Frt
LoxP
Targeted allele
F2
1b
F1
LoxP Frt
R2
LoxP
R1
Conditional KO allele
after Flp recombination
LoxP
Constitutive KO allele
after Cre recombination
B
Neg Control
WT / WT
WT / Cond KO
Cond KO / Cond KO
Neg Control
WT / WT
WT / Cond KO
Cond KO / Cond KO
bp
600
500
400
300
200
100
Primer Set F1-R1
Primer Set F2-R2

Supplementary figure 6 (related to Figure 5) | Generation of the Tpm3 gene exon 1b knockout mouse.

(A) Genomic fragments of the Exon 1b region were assembled into a targeting vector containing a Neo cassette flanked by Frt sites located upstream of exon 1b. LoxP sites were inserted upstream of the Neo cassette and in the 3' region downstream from exon 1b. A diphtheria toxin A (DTA) cassette was used in the targeting construct for negative selection. ES cells were electroporated with the targeting construct and the selected ES cell clones were microinjected into C57Bl/6 blastocysts to generate chimeras containing the targeted allele. Chimeric mice were subsequently bred against Flp transgenic mice to remove the Neo drug selection cassette via Flp-mediated recombination and obtain germline F1 mutants with the conditional knockout allele. The location of the PCR genotyping primer pairs is indicated (arrows, F1-R1 and F2-R2). Exon 1b can be excised to create the constitutive full knockout allele by Cre-mediated recombination of the LoxP sites in the conditional knockout allele.

(B) PCR genotyping of the conditional knockout allele prior to Cre-mediated recombination. Primer set 1: The wild type (WT) allele is 151 bp and the conditional knockout (Cond KO) allele is 212 bp. Primer Set 2: The wild type (WT) allele is 402 bp and the conditional knockout (Cond KO) allele is 536 bp. A negative (Neg) control for each primer set containing no template DNA is also shown. For heterozygous mice (WT/CondKO) the bands generated by the F2-R2 primer set favored the smaller sized band in abundance; whereas a more even distribution was seen with the bands generated by the F1-R1 primer set.

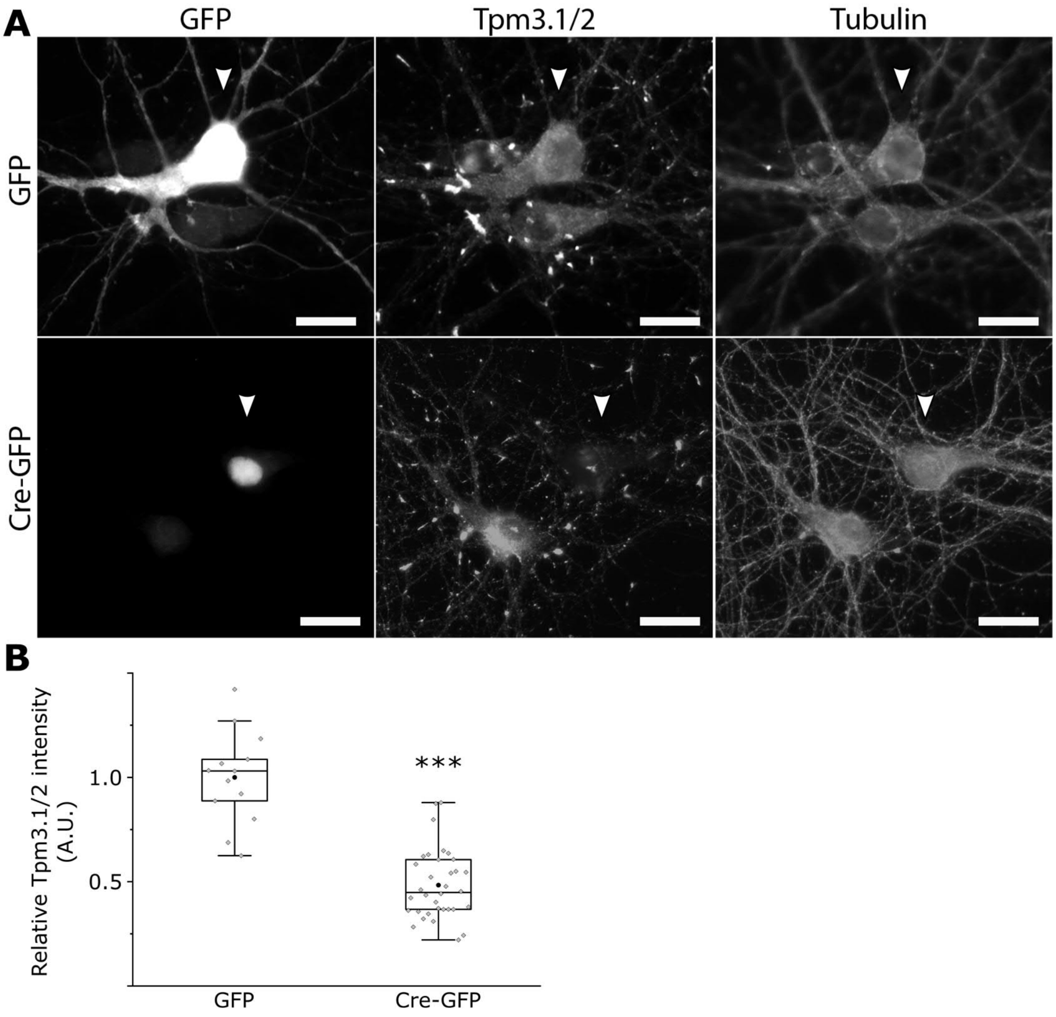

Supplementary figure 7 (related to Figure 5) | Validation of the reduction of Tpm3 protein levels.

(A) Tpm3.1/2 immunofluorescence in cultured hippocampal neurons of Tpm3 conditional knockout mice. Arrowheads indicate a neuron expressing either GFP or GFP-tagged Cre-recombinase (Cre-GFP). Scale bars: 20 μm.

(B) Normalized relative intensity of anti-2G10.2 fluorescence in the somata of neurons expressing GFP or Cre-GFP (Ratios for GFP or Cre-GFP expressing neurons relative to control neurons in the same picture, normalized to mean GFP ratio: GFP, 1.00 ± 0.06, mean ± SEM; Cre-GFP, 0.48 ± 0.03, $p < 0.001$, *t*-test). Black circles represent mean value. Box borders represent the 25th and 75th percentiles, whiskers represent minimum and maximum values less than 1.5x the interquartile range lower or higher than the 25th or 75th percentiles, respectively (Tukey style). GFP-infected: n = 13 pictures (ratios), 13 GFP-expressing cells, 21 controls; Cre-GFP infected: n = 34 pictures (ratios), 36 Cre-GFP expressing neurons, 55 controls. * denotes statistical significance. ***: $p < 0.001$.

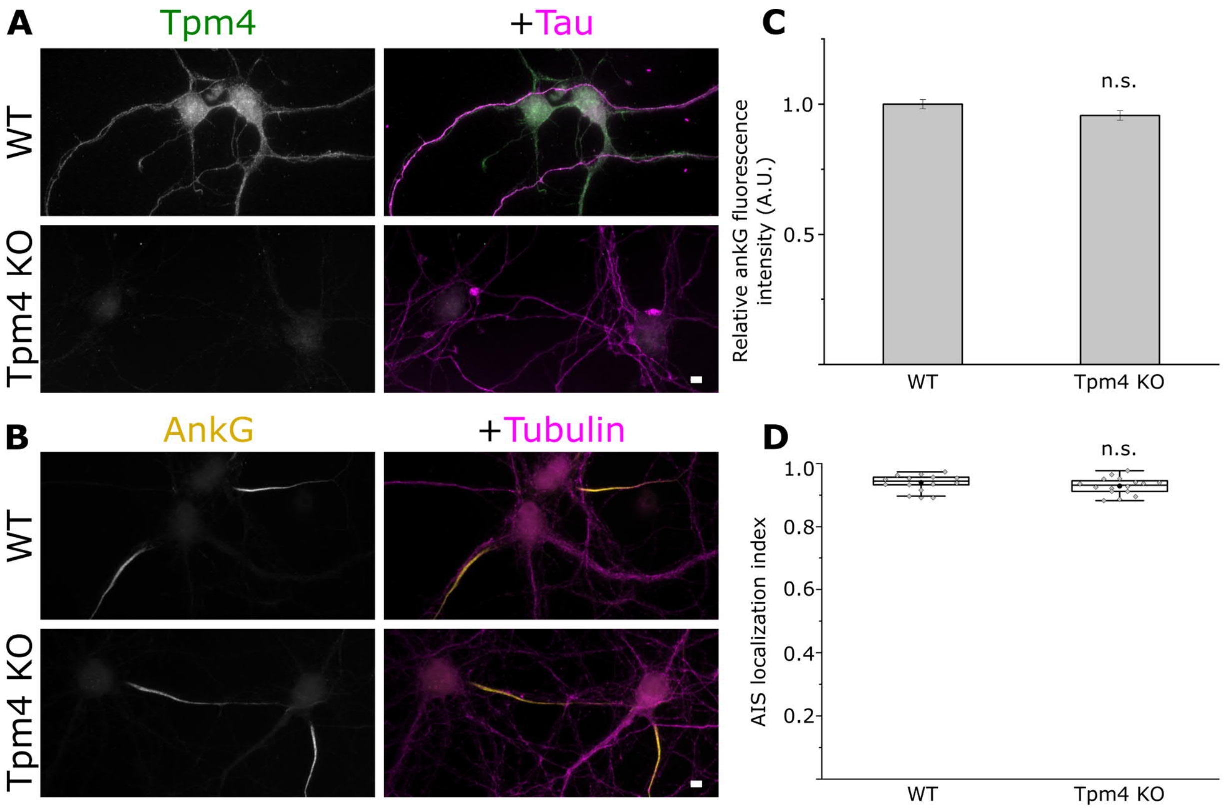

Supplementary figure 8 (related to Figure 5) | *Tpm4* knockout mice show typical accumulation of ankyrin G at the AIS.

(A) Cultured hippocampal neurons of wild-type and *Tpm4* knockout mice showing the absence of Tpm4 immunofluorescence in the knockouts. Anti-Tau served to label axons.

(B) *Tpm4* knockout mice show typical accumulation of ankyrin G. β3-tubulin served to label neurons. Scale bars: 5 µm.

(C) The difference in the mean AIS ankyrin G fluorescence intensity between wild-type (1 ± 0.02, mean ± SEM) and knock-out (0.96 ± 0.02, mean ± SEM) neurons was not statistically significant (Mann-Whitney *U* test). Wild-type: n = 122, 3 independent experiments; *Tpm4* knockout: n = 147, 3 independent experiments. Error bars represent standard error of mean.

(D) AIS localization indices (ALI) did not differ between WT and Tpm4 KO neurons (*t*-test, WT: 0.94 ± 0.01, mean ± SEM, n = 14, 2 independent experiments; Tpm4 KO: 0.93 ± 0.01, mean ± SEM, n = 16, 2 independent experiments). Black circles represent mean values. Box borders represent the 25th and 75th percentiles, whiskers represent minimum and maximum values less than 1.5x the interquartile range lower or higher than the 25th or 75th percentiles, respectively (Tukey style). n.s.: not significant.

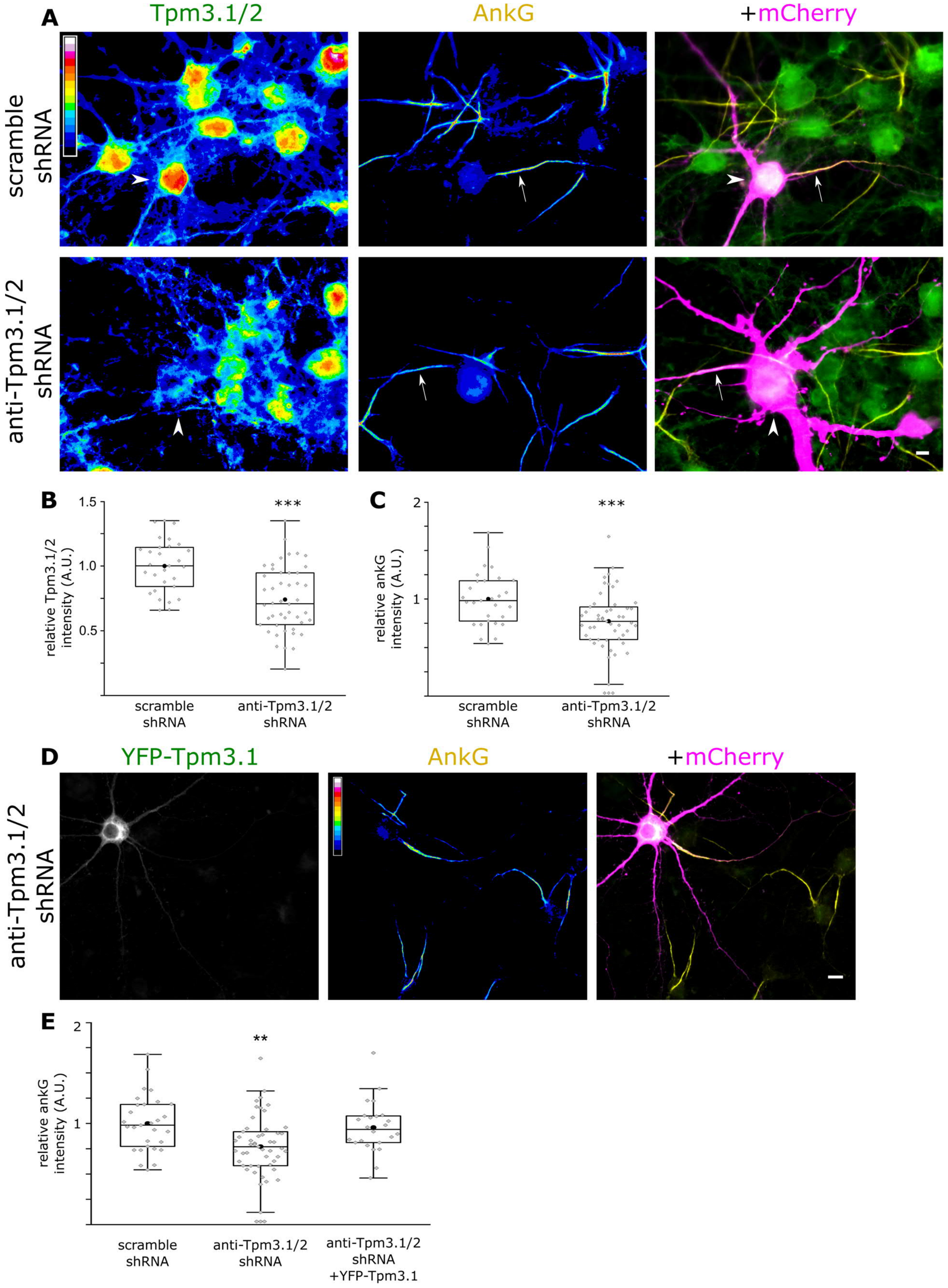
A
Tpm3.1/2
AnkG
+mCherry
scramble shRNA
anti-Tpm3.1/2 shRNA
B
relative Tpm3.1/2 intensity (A.U.)
1.5
1.0
0.5
***
scramble shRNA
anti-Tpm3.1/2 shRNA
C
relative ankG intensity (A.U.)
2
1
***
scramble shRNA
anti-Tpm3.1/2 shRNA
D
YFP-Tpm3.1
AnkG
+mCherry
anti-Tpm3.1/2 shRNA
E
relative ankG intensity (A.U.)
2
1
**
scramble shRNA
anti-Tpm3.1/2 shRNA
anti-Tpm3.1/2 shRNA +YFP-Tpm3.1

Supplementary figure 9 (related to Figure 5) | Tpm3.1/2 knockdown reduces the accumulation of ankyrin G at the AIS.

(A) Rat hippocampal neurons transfected at 10 DIV using either anti-Tpm3.1/2 or scramble shRNA, and neighboring, non-transfected neurons. Neurons were fixed at 14 DIV and stained using anti-γ/9d and anti-ankyrin G. Arrowheads point to the transfected neuron, arrows point to axons of transfected neurons. Color code indicates normalized fluorescence intensity levels. Scale bar: 5 µm.

(B) Normalized relative intensity of anti-γ/9d fluorescence in the somata of neurons expressing scramble or anti-Tpm3.1/2 shRNA (scramble shRNA: 1 ± 0.04, mean ± SEM; shRNA: 0.74 ± 0.04, mean ± SEM, $p < 0.001$, *t* test).

(C) Relative anti-ankyrin G fluorescence intensity in in each group (scramble shRNA: 1 ± 0.05, mean ± SEM; shRNA: 0.77 ± 0.04, mean ± SEM, $p < 0.01$, *t* test). Scramble shRNA: n = 29, 3 independent experiments; anti-Tpm3.1/2 shRNA: n = 51, 3 independent experiments.

(D) Rat hippocampal neurons expressing mCherry-tagged anti-Tpm3.1/2 shRNA and YFP-tagged human Tpm3.1 at 10-14 DIV. Human Tpm3.1 differs from rat Tpm3.1 in the sequence within the targeted region. Anti-ankyrin G served to visualize ankyrin G. A neuron expressing YFP-Tpm3.1 and anti-Tpm3.1/2 shRNA showing typical ankyrin G accumulation at the AIS. Color code indicates normalized fluorescence intensity levels.

(E) Relative anti-ankyrin G fluorescence intensity in in each group (scramble shRNA: 1 ± 0.05, mean ± SEM; shRNA: 0.77 ± 0.04, mean ± SEM; anti-Tpm3.1/2 shRNA + YFP-Tpm3.1: 0.96 ± 0.05, mean ± SEM; $p < 0.01$, ANOVA, Tukey's test). Anti-Tpm3.1 shRNA: n = 42, 2 independent experiments, partially used for (B) and (C); scramble shRNA: n = 28, 2 independent experiments, partially used for (B) and (C); anti-Tpm3.1/2 shRNA + YFP-Tpm3.1: n = 26, 2 independent experiments. Black circles represent mean value. Box borders represent the 25th and 75th percentiles, whiskers represent minimum and maximum values less than 1.5x the interquartile range lower or higher than the 25th or 75th percentiles, respectively (Tukey style). * denotes statistical significance. **: $p < 0.01$; ***: $p < 0.001$. Scale bar: 5 µm.

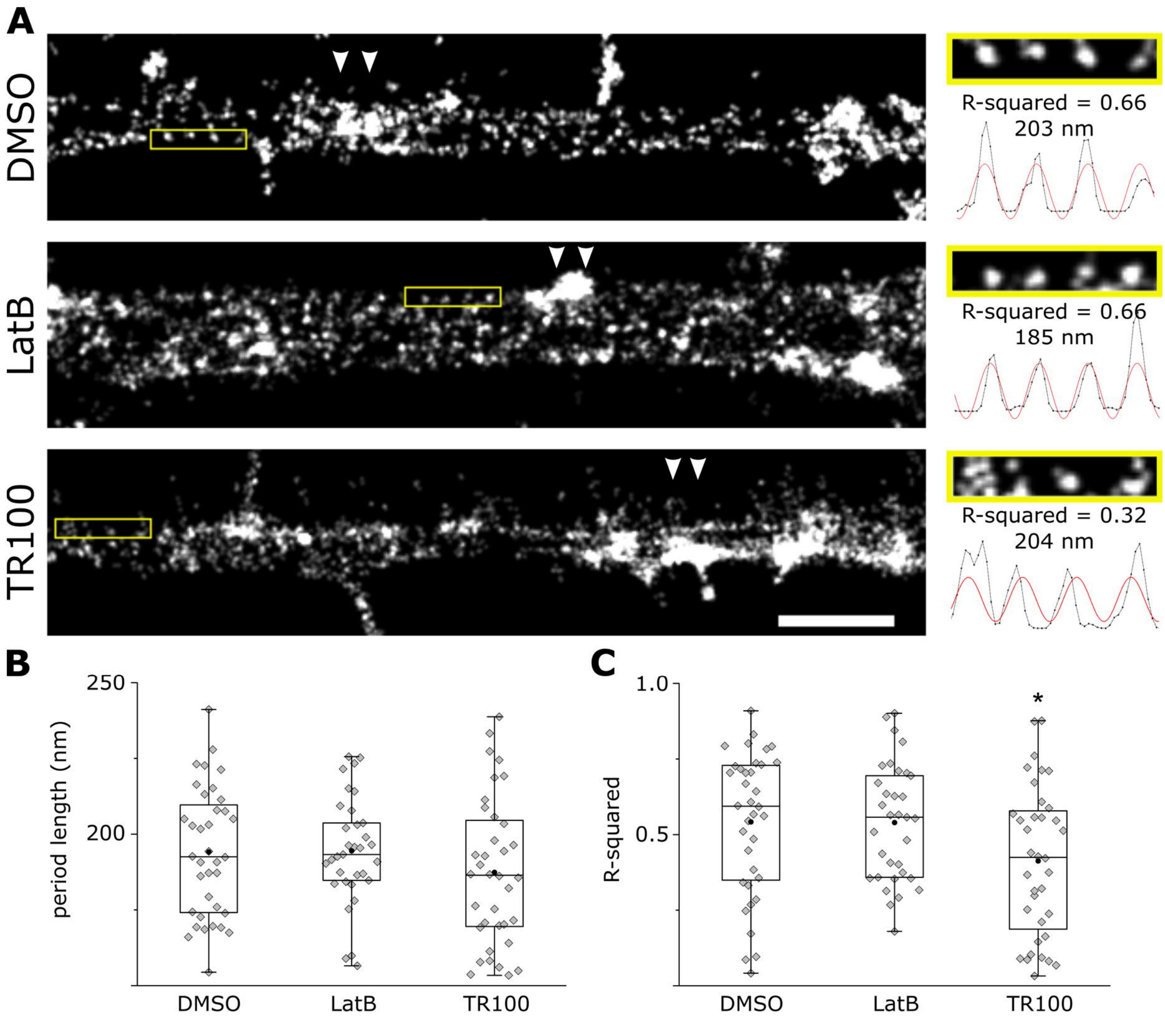

Supplementary figure 10 (related to Figure 9) | The periodicity of actin rings in the AIS is disrupted upon Tpm3.1 inhibition, but not latrunculin-B treatment.

(A) *Left*: STORM reconstructions of the AIS of rat hippocampal neurons at 14 DIV, treated overnight using either DMSO (0.2%), LatB (5 µM), or TR100 (5 µM). Alexa 647-tagged phalloidin served to label F-actin. Arrowheads point to AIS actin patches. *Right*: Higher magnification of the yellow-boxed area. The intensity profiles (black lines) and the best Fourier series fit (red lines) are shown. The period lengths and the R-squared values of the fits are indicated. 3 profiles were plotted in every neuron where periodicity was best visible.

(B) Period lengths in each group. The difference in mean period length between groups was not statistically significant (ANOVA).

(C) R-squared values of individual Fourier series fits in each group. TR100-treated neurons showed a significantly lower R-squared value, indicating a poorer Fourier series fit. LatB-treated neurons were not different from DMSO controls (Mann-Whitney *U* test). Black circles represent mean value. Box borders

represent the 25th and 75th percentiles, whiskers represent minimum and maximum values less than 1.5x the interquartile range lower or higher than the 25th or 75th percentiles, respectively (Tukey style). DMSO: n = 12 neurons, 5 independent experiments; LatB: n = 11 neurons, 4 independent experiments; TR100: n = 12 neurons, 4 independent experiments. * denotes statistical significance. *: $p < 0.05$. Scale bar: 1 µm.

TRANSPARENT METHODS

Neuronal cultures, transfections, and preparation of fixed samples

Neuronal cultures were prepared as described previously (Hotulainen et al., 2009). We collected brains from embryonic day 17 Wistar rat fetuses of either sex, removed the meninges, dissected the hippocampi, and dissociated cells in 0.05% papain. Next, we mechanically triturated and suspended the cells in Ca^{2+}- and Mg^{2+}-free HBBS medium containing sodium pyruvate (1 mM), HEPES (10 mM, pH 7.2), and DNase I (20 U/ml, Sigma-Aldrich). We plated the cells in 24-well plates on glass coverslips 13-mm in diameter coated using poly-L-lysine (0.01 mg/ml, Sigma-Aldrich) in Neurobasal medium (Invitrogen) supplemented with B-27 (Invitrogen), L-glutamine (Invitrogen), and primocin (InvivoGen). For SIM experiments, we used square high-performance glass coverslips 18 x 18 mm (Zeiss) in 6-well cell culture plates. For STORM experiments, we used glass-bottomed 35 mm dishes (Mattek). For experiments involving transfections, we plated neurons at a density of 75,000 cells/cm^2. For all other experiments, we plated 3,500 cells/cm^2. We cultured the neurons in a humidified incubator at 37°C and 5% CO_2, and refreshed the media twice weekly at regular intervals. We used Lipofectamine 2000 (Invitrogen) to transfect 8-10 DIV neurons as described previously (Hotulainen et al., 2009). We used 4% PFA in PBS for 10-15 minutes at room temperature to fix neurons in preparation for immunofluorescence. For the extraction of soluble proteins (Supplementary figure 5) we treated neurons using 0.1% Triton-X in cytoskeleton buffer (10 mM MES, 150 mM NaCl, 5 mM EGTA, 5 mM glucose, 5 mM $MgCl_2$, pH: 6.1) for 60 seconds at room temperature, followed by 4% PFA. For panNa$_v$ staining, we used 1% PFA + 3% sucrose in PBS. For EB1 staining, we treated fixed neurons using 1 mM EGTA in 100% methanol for 5 minutes at -20°C, followed by 4% PFA for 5 minutes at room temperature. We used 0.5% Triton-X in PBS for 10 minutes to permeabilize the cells and 0.2% BSA in PBS (BSA-PBS) for blocking and washing. Following primary and secondary staining, we washed the cells once in 0.1% Triton-X in PBS and 3 times in BSA-PBS. Primary and secondary antibodies were incubated in BSA-PBS at room temperature for 75 minutes and 45 minutes, respectively. For SIM experiments, we incubated γ/9d overnight at 4°C. We mounted coverslips on glass slides using Immu-Mount (Thermo/Shandon). For SIM experiments, we mounted coverslips on glass slides using ProLong Gold (ThermoFisher). Phalloidin was incubated in PBS for 2 hours at room temperature. For STORM experiments, phalloidin staining was performed immediately before imaging.

For AIS intensity experiments using Tp9, Tp16 and C57Bl6 mouse lines, hippocampal neurons were prepared as previously described (Fath et al., 2009). In brief, hippocampi were dissected from the brains of embryonic mice at embryonic day 16 (E16) and dissociated by mechanical trituration after enzymatic exposure to trypsin and DNase I (Sigma-Aldrich). Cells were plated at a density of 58,000 cells/cm^2 on PDL-coated 12 mm coverslips and maintained in neurobasal media supplemented with 2% B27 and 2 mM Glutamax (Life Technologies) at 37°C and 5% CO_2. Tp9 cultures were transduced at 0 DIV with either CMV-EGFP-Cre or CMV-EGFP adeno-associated viruses (UNC Vector Core Facility) at a concentration of 5 x 10^7 viral particles/70,000 cells. Prior to immunostaining, cultures were fixed at 9 DIV with 4% PFA in PBS for 15 min at room temperature. We then permeabilized the cells using 0.1% TritonX-100 in PBS for 5 min and blocked in 2% FBS in PBS. For Tm4 staining, cells were instead permeabilized for 5 min with ice-cold MeOH and blocked in 2% FBS in PBS. Coverslips were mounted on glass slides using ProLong Gold (Life Technologies).

Plasmids, antibodies and reagents

mCherry-C1 (mCherry) was purchased from Clontech. PAGFP-actin (Dopie et al., 2012) was a kind gift from Maria Vartiainen (University of Helsinki, Finland). YFP-Tpm3.1 and YFP-Tpm3.2 were described previously (Tojkander et al., 2011). shRNA against rat Tpm3.1 (NM_173111.1) was purchased from GeneCopoeia (RSH053175-33-mH1, target sequence CCAAGTCTTAGCCAAACAACA). Mouse monoclonal anti-ankyrin G antibody (1:1000, UC Davis/NIH NeuroMab Facility, Clone 106/36) and mouse monoclonal panNF-186 (1:500, UC Davis/NIH NeuroMab Facility, Clone A12/18) were purchased from NeuroMab. Sheep polyclonal anti-γ/9d (1:100, for SIM: 1:50, AB5447), mouse monoclonal anti-Tpm3 (clone 2G10.2, MAB2256), rabbit polyclonal anti-GluA1 (1:300, Chemicon/Millipore AB1504), chicken polyclonal anti-MAP2 (1:10000, AB5543), chicken anti-β3 tubulin (1:500, AB9354) and mouse anti-ankyrin G (1:500, MABN466) were purchased from Merck Millipore. Rabbit polyclonal anti-panNa$_v$ was purchased from Alomone Labs (1:200, ASC-003). Rabbit anti-myosin IIB (1:500) was purchased from Australian Biosearch (909901). Rabbit anti-TRIM46 (1:100) was described previously (van Beuningen et al., 2015). Mouse anti-EB1 (1:100, 610535) were purchased from BD Transduction Lab. Rabbit anti-GFP (1:1000, AB290) was purchased from Abcam. Rabbit polyclonal δ9d (1:250, purified serum) was produced by the Gunning lab and previously described

(Schevzov et al., 2011). Alexa 647- and Alexa 488-conjugated phalloidin were purchased from ThermoFisher (1 μM, A22287). Alexa Fluor-conjugated secondary antibodies (1:400) were purchased from ThermoFisher. The anti-tropomyosin drugs TR100 and Anisina (ATM3507) were described previously (Currier et al., 2017; Stehn et al., 2016; Stehn et al., 2013) and were added to culture media from 50-mM stock solutions in DMSO. Latrunculin B (Sigma, L5288) was added to culture media from a 2.5-mM stock solution in DMSO.

Generation of the Tpm3 gene exon 1b conditional knockout mouse

Genomic fragments of the *Tpm3* exon 1b region were assembled into a targeting vector containing a Neo cassette flanked by Frt sites located upstream of exon 1b (Supplementary figure 9). LoxP sites were inserted upstream of the Neo cassette and in the 3' region downstream from exon 1b. A diphtheria toxin A (DTA) cassette was used in the targeting construct for negative selection. ES cells were electroporated with the targeting construct and the selected ES cell clones were microinjected into C57Bl/6 blastocysts to generate chimeras containing the targeted allele. Chimeric mice were subsequently bred against Flp transgenic mice to remove the Neo drug selection cassette via Flp-mediated recombination and obtain germline F1 mutants with the conditional knockout allele. Exon 1b can be excised to create the constitutive full knockout allele by Cre-mediated recombination of the LoxP sites in the conditional knockout allele. The conditional knockout allele prior to Cre-mediated recombination was tested by PCR genotyping (Supplementary figure 9b).

Imaging

For taking confocal stacks, we used a Zeiss LSM880 inverted confocal microscope (Zeiss) equipped with a 63x 1.40 NA oil-immersion objective or a Zeiss LSM710 upright confocal microscope (Zeiss) equipped with a 63x 1.46 NA oil-immersion objective. Both microscopes were equipped with 405, 488, 561, and 633 nm laser lines. We adjusted laser power and gain settings as to maximize signal-to-noise ratio while ensuring no pixels were saturated outside the somata. *Z* stacks were acquired at a step size of 0.2 μm. Imaging of fixed samples was performed at room temperature. For live-cell imaging we used a temperature-controlled chamber and CO_2 supply. We imaged live cells in culture media at 37°C and 5% CO_2 using a Zeiss LSM710 upright confocal microscope equipped with a 63x 1.0 NA water-dipping objective. For epifluorescence imaging,

we used a Zeiss Axio Imager Z2 upright epifluorescence microscope (Zeiss) equipped with a 40x 1.3 NA oil-immersion objective and a Hamamatsu Orca Flash 4.0 LT camera (Hamamatsu). Alternatively, we used a Zeiss Axio Imager M1 upright epifluorescence microscope (Zeiss) equipped with a 20x 0.8 NA objective and an AxioCam HRm camera (Zeiss). We used Zeiss Zen software for acquisition.

For SIM, we used a DeltaVision OMX SR imaging system (GE Healthcare Life Sciences) equipped with a 60x 1.42 NA PlanApo N oil objective, 488, 560, and 640 nm laser lines, and 3 sCOMS cameras. We used the AcquireSR software for acquisition and SoftWorx software for image reconstruction and alignment.

For STORM, we used an N-STORM system comprising a Nikon Eclipse Ti-E inverted microscope with Nikon IR-based Perfect Focus System (Nikon Instruments). The microscope was equipped with a 100x 1.49 NA Apo TIRF oil-immersion objective, 405 and 647 nm laser lines (100 and 300 mW, respectively), and an iXon+ 897 camera (Andor). We used a Nikon Intensilight metal arc light source (Nikon) to locate neurons. We used NIS-Elements software for acquisition. We placed the neurons in STORM buffer immediately before imaging: pH 7.9 50 mM Tris, 10% glucose, 70 mM freshly prepared MEA (Sigma), 0.75 mg/ml glucose oxidase (Sigma), and 0.04 mg/ml catalase (Sigma). We continuously illuminated the sample using the 647-nm laser at full power for a series of 20,000-30,000 images of 20-40 ms exposure time (128 x 128 pixels). We used an increasing intensity of 405-nm illumination to reactivate fluorophores. For the analysis of the AIS in mouse hippocampal neurons, fixed samples were imaged on an Axioskop 40 microscope (Zeiss) using a 40x oil objective. AIS intensity was measured using ImageJ software and statistically analyzed in GraphPad Prism (v7.02).

Photoactivation

For photoactivation experiments, live neurons were stained using panNF-186 to label the AISs (Hedstrom et al., 2008). 1-2 hours before imaging, neurons were incubated in culture media containing panNF-186 for 10 minutes at 37°C and 5% CO_2, washed 3 times in Neurobasal media, then incubated in culture media containing anti-mouse Alexa Fluor-647 for 10 minutes at 37°C and 5% CO_2. Neurons were then washed and returned to culture media. We acquired a frame every 3 seconds covering an area of 38.4 x 38.4 μm (256 x 256 pixels) with a pixel dwell time of 3.15 μs. After acquiring 3 pre-activation frames, we used 10-15 iterations of a 405-nm laser at full

power (30 mW) to induce photoactivation. To measure the rate of fluorescence decay we limited the photoactivation to a square region of interest 2.25 x 2.25 µm (15 x 15 pixels). Acquisition was resumed immediately after photoactivation for 360 s at 3-s intervals.

For each experiment, the fluorescence intensity within the region of interest in the pre-activation frames was measured, averaged, and the value was subtracted from subsequent measurements. The first post-activation frame was taken as time-point 0 s. The average fluorescence intensity in subsequent frames (F_t) were normalized to the first frame (F_0) to plot fluorescence decay curves. Decay curves were fit to the two-component exponential function $F(x) = F_1 . e^{\frac{-x}{t1}} + F_2 . e^{\frac{-x}{t2}}$ where x is time, F_1 is the component with the smaller time constant (dynamic pool), F_2 is the component with the larger time constant (stable pool), and t_1 and t_2 are the respective time constants (Star et al., 2002). The proportions of the dynamic and stable pools were calculated as $F_d = \frac{F_1}{F_1+F_2}$ and $F_s = \frac{F_2}{F_1+F_2}$, respectively.

Electrophysiology

For electrophysiological recordings, we placed coverslips in a submerged recording chamber and perfused using an extracellular solution containing (in mM): 124 NaCl, 3 KCl, 1.25 NaH_2PO_4, 1 $MgSO_4$, 26 $NaHCO_3$, 15 D-glucose, 2 $CaCl_2$; bubbled using 5% CO_2/95% O_2 at 32°C. We used patch electrodes (3-5 MΩ) to perform whole-cell recordings in individual cells. Whole-cell pipettes used for current-clamp experiments contained the following (in mM): 130 K-gluconate, 8 NaCl, 10 HEPES, 0.4 EGTA, 4 Mg-ATP and 0.3 Na-GTP, with the addition of either Anisina (2.5 µM) or DMSO (0.2%). The osmolarity of all intracellular solutions was adjusted to 285 mOsm and the pH to 7.25. Action potentials were detected and analyzed using the Mini Analysis Program 5.6.6. (Synaptosoft). Firing frequency was calculated from the interval between the second and the third action potential during a 500 ms depolarizing step evoked by 100-200 pA. Data are expressed as frequency. The phase plane plots were constructed with Clampex software using the first derivative of the somatic membrane potential (dV/dt) versus the somatic membrane potential. Action potential threshold was determined from the first inflection point (here, when the phase plane plot reached 10mV/ms (Naundorf et al., 2006)).

Image Analysis

We used the Fiji software platform for image analysis (Schindelin et al., 2012). We plotted the fluorescence intensity profile ~1-5 μm along the AIS where periodicity was visible in a single plane in a SIM reconstruction, avoiding patches and fasciculations. We used a MATLAB script to locally normalize fluorescence intensity in each profile. We used the "autocorr" function in MATLAB to calculate the autocorrelation function for each profile and obtain autocorrelation curves. We used the "findpeaks" function in MATLAB to detect individual peaks in each profile and note their locations to calculate inter-peak distances.

To quantify the effect of Tpm3.1/2 shRNA knockdown on the accumulation of ankyrin G at the AIS, we used NeuronJ (Meijering et al., 2004) to blindly trace the AISs of transfected neurons expressing either scramble or anti-Tpm3.1/2 shRNA, as well as neighboring non-transfected neurons. We used a 3-μm moving average to smooth fluorescence intensity profiles (van Beuningen et al., 2015) and noted the peak intensity of every profile. Relative ankyrin G fluorescence intensity was calculated by comparing the peak intensity recorded in the transfected neuron to the mean peak intensity of the neighboring non-transfected neurons. We then normalized the mean values to the mean relative intensity of neurons expressing scramble shRNA.

Figure 5B: AIS intensity was measured using the line tool in ImageJ (v1.51s). Briefly, a segmented line was traced along the thickest length of the AIS as indicated by the Ankyrin-G fluorescence signal. ImageJ then measured the signal intensity and length of the area associated with the segmented line. Raw data obtained from ImageJ were exported to excel then GraphPad Prism (v7.02) for statistical analyses.

Figure 10: The AIS region was determined using an Ankyrin G antibody (cat. #MABN466, Merck). Using the corresponding Ankyrin G signal, the fluorescence intensity of myosin IIB (cat. #909901, Australian Biosearch) was measured along the initial 20 μM of the AIS. Image J software was used to measure fluorescence intensity.

For calculating the ALI, we measured the fluorescence intensity profile of either anti-ankyrin G or panNa$_v$ in the initial 30 μm of every neurite in a single neuron. After subtracting background fluorescence, we calculated the 3-μm moving average to smooth the profiles (van Beuningen et al., 2015), trimmed the profiles for even averaging, and then normalized all measurements to the peak intensity value. We then noted the normalized peak value for each neurite and used them to

calculate the median peak value. AIS localization index was expressed as $ALI = 1 - median_{peaks}$ such that a value closer to 1 indicates the strongest accumulation in a single neurite, while a value closer to 0 indicates homogenous concentration across all neurites.

For calculating the ADR, we used NeuronJ (Meijering et al., 2004) to trace the axon and dendrites in confocal stacks. We then used a maximum intensity projection to measure background fluorescence, the mean fluorescence intensity along ~100 μm of the axon distal to the AIS, and the mean fluorescence along all dendrites. ADR was calculated as $ADR = \frac{(F_{axon} - F_{background})}{(F_{dendrites} - F_{background})}$ (Lewis et al., 2009).

We used custom scripts written by Leterrier et al. (2015) to convert STORM localization files into a format readable by the ThunderSTORM plugin (Ovesny et al., 2014), which was used for reconstructing images. For evaluating periodicity, we used MATLAB to fit intensity profiles to a single-term Fourier series represented by $y = a_0 + a_i \cos(iwx) + b_i \sin(iwx)$ and obtained the period length and the R-squared value reflecting goodness of fit.

Colocalization analysis between F-actin (Alexa-488) and Tpm3.1/2 (Alexa 647) -positive signals was performed on maximum intensity projections of SIM images. Importantly, SIM imaging resulted in a slight variability in Z-levels for different fluorophores and therefore based on a visual inspection three consecutive Z-layers at best focus for each fluorophore were manually selected to be included in the maximum intensity projection of a Z-stack. To estimate the error that the Z-level shift (or any technical aspect depending on different fluorophores) gives for the colocalization quantification, we performed the same experiment having double Tpm3.1/2 stainings visualized with 488- and 647-dyes. AIS region was selected for the analysis based on ankyrin G staining (Alexa-594). Regions of interest (ROIs) were hand-drawn outlining the AIS regions.

The 'EzColocalization' Fiji/ImageJ plugin was used for colocalization analysis in ROIs of maximum intensity projections according to the protocol described by (Stauffer et al. 2018). Briefly, each fluorophore channel was subject to automatic thresholding to remove background, and the Pearson correlation coefficient (PCC) was calculated to get a value between -1 and 1, reflecting the degree of co-occurrence of signal intensities between both F-actin and Tpm3.1/2. The value 1 indicates perfectly linearly related intensities, the value −1 perfectly inversely linearly

related intensities and the values near 0 intensities that are uncorrelated with one another (Dunn et al., 2011).

Statistical Analyses

Binomial tests were performed using the SPSS software package. All other statistical analyses were performed using the OriginLab software package. Intensity profiles curves for STORM data were created using MATLAB. All other graphs were created using OriginLab. Data were checked for normality (Shapiro-Wilk test) and homogeneity of variance (Levene's test). For parametric data, we used the independent two-sample *t* test, or one-way ANOVA and Tukey's test for post-hoc analysis. For non-parametric data, we used Kruskal-Wallis ANOVA and Mann-Whitney *U* Test. Bonferroni correction for multiple comparisons was used wherever appropriate. We used Kolmogorov-Smirnov test to compare the distributions of inter-peak distances under different conditions. We created box plots in Tukey style (Krzywinski and Altman, 2014) with the addition of solid black circles to represent the mean. Classification of photoactivation data as belonging to actin patches or areas outside the patches was done using hierarchical cluster analysis. We used double-exponential decay fits of 44 normalized fluorescence decay curves recorded from the AIS, each responding to 120 variables corresponding to fluorescence levels at 120 time points between 0 and 357. This number of time points corresponds to the sampling frequency of the recordings. We ran a hierarchical cluster analysis using Ward's method (squared Euclidean distance, data standardization: range 0-1) that assigned 15 curves as belonging to AIS actin patches, and 29 curves as recorded from areas outside the patches. To test the significance of the difference between the resultant clusters, we performed one-way ANOVA for each time point. The between-groups difference was significant at all time points after 0 s ($p < 0.05$). The difference between recordings from dendrites and areas in the AIS outside the actin patches was not significant at any time point (Tukey's test), while recordings from the patches were significantly different from recordings from both dendrites and areas outside the patches (Tukey's test, $p < 0.05$) for all time points after 0 s.

SUPPLEMENTAL REFERENCES

Currier, M.A., Stehn, J.R., Swain, A., Chen, D., Hook, J., Eiffe, E., Heaton, A., Brown, D., Nartker, B.A., Eaves, D.W., et al. (2017). Identification of Cancer-Targeted Tropomyosin Inhibitors and Their Synergy with Microtubule Drugs. Mol Cancer Ther 16, 1555-1565.

Dopie, J., Skarp, K.P., Rajakyla, E.K., Tanhuanpaa, K., and Vartiainen, M.K. (2012). Active maintenance of nuclear actin by importin 9 supports transcription. Proc Natl Acad Sci U S A 109, E544-552.

Dunn, K.W., Kamocka, M.M., and McDonald, J.H. (2011). A practical guide to evaluating colocalization in biological microscopy. Am J Physiol Cell Physiol 300, C723-742.

Fath, T., Ke, Y.D., Gunning, P., Gotz, J., and Ittner, L.M. (2009). Primary support cultures of hippocampal and substantia nigra neurons. Nat Protoc 4, 78-85.

Hotulainen, P., Llano, O., Smirnov, S., Tanhuanpaa, K., Faix, J., Rivera, C., and Lappalainen, P. (2009). Defining mechanisms of actin polymerization and depolymerization during dendritic spine morphogenesis. J Cell Biol 185, 323-339.

Krzywinski, M., and Altman, N. (2014). Points of Significance: Visualizing samples with box plots. Nat Meth 11, 119-120.

Leterrier, C., Potier, J., Caillol, G., Debarnot, C., Rueda Boroni, F., and Dargent, B. (2015). Nanoscale Architecture of the Axon Initial Segment Reveals an Organized and Robust Scaffold. Cell Rep 13, 2781-2793.

Lewis, T.L., Jr., Mao, T., Svoboda, K., and Arnold, D.B. (2009). Myosin-dependent targeting of transmembrane proteins to neuronal dendrites. Nat Neurosci 12, 568-576.

Meijering, E., Jacob, M., Sarria, J.C., Steiner, P., Hirling, H., and Unser, M. (2004). Design and validation of a tool for neurite tracing and analysis in fluorescence microscopy images. Cytometry A 58, 167-176.

Ovesny, M., Krizek, P., Borkovec, J., Svindrych, Z., and Hagen, G.M. (2014). ThunderSTORM: a comprehensive ImageJ plug-in for PALM and STORM data analysis and super-resolution imaging. Bioinformatics 30, 2389-2390.

Schevzov, G., Whittaker, S.P., Fath, T., Lin, J.J., and Gunning, P.W. (2011). Tropomyosin isoforms and reagents. Bioarchitecture 1, 135-164.

Stauffer, W., Sheng, H., and Lim, H.N. (2018). EzColocalization: An ImageJ plugin for visualizing and measuring colocalization in cells and organisms. Sci Rep 8, 15764.

Stehn, J., Mariana, A., Failes, T., Ashokakumar, V., Eiffe, E., Heaton, A., Hook, J., Sivanandhan, D., Arndt, G., Gunning, P., et al. (2016). Synergistic action of first-in-class anti-tropomyosin compound, ATM-3507 (Anisina) and microtubule targeting inhibitors in pre-clinical models of non-small cell lung cancer (NSCLC). European Journal of Cancer 69, S116.

Stehn, J.R., Haass, N.K., Bonello, T., Desouza, M., Kottyan, G., Treutlein, H., Zeng, J., Nascimento, P.R., Sequeira, V.B., Butler, T.L., et al. (2013). A novel class of anticancer compounds targets the actin cytoskeleton in tumor cells. Cancer Res 73, 5169-5182.

Tojkander, S., Gateva, G., Schevzov, G., Hotulainen, P., Naumanen, P., Martin, C., Gunning, P.W., and Lappalainen, P. (2011). A molecular pathway for myosin II recruitment to stress fibers. Curr Biol 21, 539-550.

van Beuningen, S.F., Will, L., Harterink, M., Chazeau, A., van Battum, E.Y., Frias, C.P., Franker, M.A., Katrukha, E.A., Stucchi, R., Vocking, K., et al. (2015). TRIM46 Controls Neuronal Polarity and Axon Specification by Driving the Formation of Parallel Microtubule Arrays. Neuron 88, 1208-1226.
